# The Human Pancreatic Islet Transcriptome: Expression of Candidate Genes for Type 1 Diabetes and the Impact of Pro-Inflammatory Cytokines

**DOI:** 10.1371/journal.pgen.1002552

**Published:** 2012-03-08

**Authors:** Décio L. Eizirik, Michael Sammeth, Thomas Bouckenooghe, Guy Bottu, Giorgia Sisino, Mariana Igoillo-Esteve, Fernanda Ortis, Izortze Santin, Maikel L. Colli, Jenny Barthson, Luc Bouwens, Linda Hughes, Lorna Gregory, Gerton Lunter, Lorella Marselli, Piero Marchetti, Mark I. McCarthy, Miriam Cnop

**Affiliations:** 1 Laboratory of Experimental Medicine, Université Libre de Bruxelles (ULB), Brussels, Belgium; 2 Functional Bioinformatics (FBI), Centre Nacional d'Anàlisi Genòmica (CNAG), Barcelona, Spain; 3 Cell Differentiation Unit, Diabetes Research Centre, Vrije Universiteit Brussel (VUB), Brussels, Belgium; 4 Oxford Centre for Diabetes, Endocrinology, and Metabolism (OCDEM), Churchill Hospital, Oxford, United Kingdom; 5 Department of Endocrinology and Metabolism, University of Pisa, Pisa, Italy; 6 Wellcome Trust Centre for Human Genetics, University of Oxford, Oxford, United Kingdom; 7 Oxford NIHR Biomedical Research Centre, Churchill Hospital, Oxford, United Kingdom; 8 Division of Endocrinology, Erasmus Hospital, Université Libre de Bruxelles (ULB), Brussels, Belgium; Georgia Institute of Technology, United States of America

## Abstract

Type 1 diabetes (T1D) is an autoimmune disease in which pancreatic beta cells are killed by infiltrating immune cells and by cytokines released by these cells. Signaling events occurring in the pancreatic beta cells are decisive for their survival or death in diabetes. We have used RNA sequencing (RNA–seq) to identify transcripts, including splice variants, expressed in human islets of Langerhans under control conditions or following exposure to the pro-inflammatory cytokines interleukin-1β (IL-1β) and interferon-γ (IFN-γ). Based on this unique dataset, we examined whether putative candidate genes for T1D, previously identified by GWAS, are expressed in human islets. A total of 29,776 transcripts were identified as expressed in human islets. Expression of around 20% of these transcripts was modified by pro-inflammatory cytokines, including apoptosis- and inflammation-related genes. Chemokines were among the transcripts most modified by cytokines, a finding confirmed at the protein level by ELISA. Interestingly, 35% of the genes expressed in human islets undergo alternative splicing as annotated in RefSeq, and cytokines caused substantial changes in spliced transcripts. Nova1, previously considered a brain-specific regulator of mRNA splicing, is expressed in islets and its knockdown modified splicing. 25/41 of the candidate genes for T1D are expressed in islets, and cytokines modified expression of several of these transcripts. The present study doubles the number of known genes expressed in human islets and shows that cytokines modify alternative splicing in human islet cells. Importantly, it indicates that more than half of the known T1D candidate genes are expressed in human islets. This, and the production of a large number of chemokines and cytokines by cytokine-exposed islets, reinforces the concept of a dialog between pancreatic islets and the immune system in T1D. This dialog is modulated by candidate genes for the disease at both the immune system and beta cell level.

## Author Summary

Pancreatic beta cells are destroyed by the immune system in type 1 diabetes mellitus, causing insulin dependence for life. Candidate genes for diabetes contribute to this process by acting both at the immune system and, as we suggest here, at the pancreatic beta cell level. We have utilized a novel technology, RNA sequencing, to define all transcripts expressed in human pancreatic islets under basal conditions and following exposure to cytokines, pro-inflammatory mediators that contribute to trigger diabetes. Our observations double the number of known genes present in human islets and indicate that >60% of the candidate genes for type 1 diabetes are expressed in beta cells. The data also show that pro-inflammatory cytokines modify alternative splicing in human islets, a process that may generate novel RNAs and proteins recognizable by the immune system. This, taken together with the findings that pancreatic beta cells themselves express and release many cytokines and chemokines (proteins that attract immune cells), indicates that early type 1 diabetes is characterized by a dialog between beta cells and the immune system. We suggest that candidate genes for diabetes function at least in part as “writers” for the beta cell words in this dialog.

## Introduction

Type 1 diabetes (T1D) is an autoimmune disease with a strong genetic component [1]. We have previously proposed that insulitis, the pancreatic islet inflammation present in T1D, results from a “dialog” between immune cells homing into the islets and the target beta cells. Beta cells contribute to this dialog by local release of cytokines and chemokines and by delivering immunogenic signals during the cell death process; this, together with signals generated by invading immune cells, contributes to trigger and amplify (or dampen) insulitis [2]. The amplification or resolution of insulitis, and its progression or not to disease, probably depends on an interplay between environmental triggers, such as dietary components or viral infections, and the patient's genetic background [2], [3], [4] acting at least in part at the pancreatic beta cell level [5], [6], [7]. It is thus important to identify the molecular mechanisms by which immune signals and genetic and/or environmental factors affect beta cell survival and the production of inflammatory mediators such as chemokines and cytokines.

Evaluation of the full transcriptome of beta cells exposed to pro-inflammatory cytokines such as interleukin-1β (IL-1β), tumor necrosis factor-α (TNF-α) and interferon-γ (IFN-γ) provides a snapshot of the responses of these cells under conditions that may prevail in early T1D [2]. Until recently, the only way to analyze large numbers of transcripts was via oligonucleotide array technology. By using this technology we have described expression of nearly 8,000 genes in rat and human islet cells, of which around 20% were modified by cytokines [8], [9], [10]. Arrays, however, can only identify known transcripts due to the need for complementary recognition of probes by the target mRNA. In recent years RNA-sequencing (RNA-seq) has emerged as a new and promising tool for transcriptomic studies. RNA-seq works in an unbiased way, without the need for *a priori* knowledge of the targets, and shows both high reproducibility and low frequency of false positives [11], [12]. Moreover, RNA-seq is able to identify between 25 and 75% more genes than cDNA microarrays, and allows identification of both whole genes and splice variants [12], [13], [14].

Transcripts of >90% of eukaryotic genes can undergo alternative splicing (AS), i.e. be spliced in more than one way [15]. AS is a basic mechanism for the generation of multiple structurally and functionally distinct mRNAs and protein isoforms from a single gene [15], [16], [17]. It varies in a tissue-specific manner, contributing to tissue specificity [18], [19], [20], and can be modulated by cellular signals such as those provided by pro-inflammatory cytokines [9]. The use of RNA-seq, coupled to dedicated bioinformatic tools, enables the identification of novel splice variants by transcripts with skipped exons, retained introns, alternative start sites, etc [16].

Against this background, we describe here the first RNA-seq analysis of human pancreatic islets. This was done by reverse transcribing and sequencing RNA from human islets obtained from five organ donors, exposed or not to the pro-inflammatory cytokines IL-1β and IFN-γ. The data showed very good internal consistency, and allowed us:

1. To describe the complete human islet cell transcriptome, including splice variants, which provides a novel and valuable resource for future genetic and functional studies;
2. To show that >60% of the candidate genes for T1D, previously believed to be mostly expressed in the immune system [21], are expressed in human islets, and that expression of many of these genes is modified by cytokines;
3. To characterize the impact of an inflammatory challenge, i.e. exposure to pro-inflammatory cytokines, on the human islet transcriptome;
4. To validate some of the key findings obtained by RNA-seq by other methods, e.g. real time RT-PCR, ELISA or histology, in independent samples of human islets and clonal or primary rat beta cells. For some of the novel genes, the use of specific siRNAs allowed clarification of their function in beta cells.

## Methods

### Ethics statement

Human islet collection and handling were approved by the local Ethical Committee in Pisa, Italy. Wistar rats were used according to the rules of the Belgian Regulations for Animal Care with approval of the Ethical Committee for Animal Experiments of the ULB.

### Human islet isolation and culture and rat beta cell culture

Human islet preparations were obtained in collaboration with Pisa University [5], [22], [23], [24]. The donors, aged 68±3 (n = 15), were heart-beating organ donors with no medical history of diabetes or metabolic disorders. Donor information is summarized in Table 1. Preparations 1–5 were used for RNA-seq and preparations 6–15 for independent confirmation of key findings. Isolated islets were used for research when the pancreas was not suitable for clinical transplantation. The human islets were isolated using collagenase digestion and density gradient purification [25]. The islets were cultured in M199 culture medium containing 5.5 mM glucose and shipped within 1–5 days following isolation. Upon arrival, the human islet cells were cultured in Ham's F-10 medium containing 6.1 mM glucose, 10% fetal bovine serum (FBS), 2 mM GlutaMAX, 50 µM 3-isobutyl-1-methylxanthine, 1% BSA, 50 U/ml penicillin and 50 µg/ml streptomycin. The islets were exposed or not to cytokines in the same medium without FBS for 2 days [5], [26]. The following cytokine concentrations were used, based on previous dose-response experiments from our group [5], [27], [28]: recombinant human IL-1β (specific activity 1.8×10^7^ U/mg; a kind gift from C.W. Reinolds, National Cancer Institute, Bethesda, MD, USA) at 50 U/ml; recombinant human IFN-γ (specific activity 2×10^7^ U/mg; R&D Systems, Abingdon, UK) at 1000 U/ml. The evaluation of islet cell purity, i.e. the percentage of beta cells present in the preparations, was done by immunocytochemistry with an anti-insulin antibody (1/1000; Sigma, Bornem, Belgium) and donkey anti-mouse IgG rhodamine (1/200; Lucron Bioproducts, De Pinte, Belgium). Only preparations with more than 40% beta cells were used for the RNA-seq analyses; on average they contained 58% beta cells (Table 1), which is similar to the reported percentage of 54% in isolated human islets [29] and 55% in the human pancreas [30].

**Table 1:** Characteristics of the organ donors and human islet preparations used for RNA-seq and independent confirmation.

		Gender	Age (years)	BMI (kg/m^2^)	Cause of death	Purity (%)
Islets for RNA-seq	ID1	F	77	24	trauma	45
	ID2	F	46	23	CVD	60
	ID3	F	79	28	trauma	61
	ID4	M	36	26	CVD	62
	ID5	M	77	25	CVD	62
Islets for RT-PCR	ID6	M	59	25	trauma	70
	ID7	F	84	26	CH	73
	ID8	M	83	24	CH	52
	ID9	F	70	25	CH	63
	ID10	M	68	37	CH	57
	ID11	M	69	24	CVD	57
	ID12	M	70	21	CVD	69
	ID13	M	75	28	CVD	59
	ID14	M	58	25	CH	59
	ID15	F	72	24	CH	62

ID: Donor identification number; F: Female; M: Male; BMI: Body mass index; CVD: Cardiovascular disease; CH: Cerebral hemorrhage. Purity indicates the percentage of beta cells in the human islet preparations as determined by staining for insulin.

For confirmation and mechanistic studies of selected genes, we used the rat insulin-producing INS-1E cell line, kindly provided by C. Wollheim, University of Geneva, Geneva, Switzerland [31]. The cells were maintained in RPMI 1640 medium supplemented with 5% heat-inactivated FBS, 10 mM HEPES, 1 mM Na-pyruvate and 50 µM 2-mercaptoethanol [26]. Cells were exposed to 10 U/ml human IL-1β and 100 U/ml murine IFN-γ (R&D Systems). These cytokine concentrations were selected based on previous dose-response studies [28], [32]; lower cytokine concentrations and shorter time points were used for rodent experiments because rat beta cells are more sensitive than human islets to cytokine damage [33], [34]. Additional confirmation was done in autofluorescence-activated cell sorting (FACS)-purified primary rat beta cells. Pancreatic islets were isolated from adult male Wistar rats (Charles River Laboratories, Brussels, Belgium) and primary beta cells FACS-purified (FACSAria; BD Bioscience, San Jose, CA, USA) and cultured as described [35]. Primary beta cells were transfected with the synthetic double-stranded (ds) RNA polyinosinic-polycytidylic acid (PIC, InvivoGen) as described [6], [7].

### RNA sequencing

Five human islet preparations were used for sequencing. Total RNA was isolated using the RNeasy Mini Kit (Qiagen, Venlo, The Netherlands) which favors purification of all RNA molecules longer than 200 nucleotides and sample preparation done as described by the manufacturer (Illumina, Eindhoven, The Netherlands). Briefly, mRNA was purified from two µg total RNA using oligo (dT) beads, before it was fragmented and randomly primed for reverse transcription followed by second-strand synthesis to create ds cDNA fragments. The generated cDNA had undergone paired-end repair to convert overhangs into blunt ends. After 3′-monoadenylation and adaptor ligation, cDNAs were purified on a 2% agarose gel and 200 basepair (bp) products were excised from the gel. Following gel digestion, purified cDNA was amplified by PCR using primers specific for the ligated adaptors. The generated libraries were submitted to quality control with the Agilent bioanalyzer 2100 (Agilent Technologies, Wokingham, UK) before sequencing. The RNA integrity number (RIN) values for all samples were 7.5 and above. 1 µL cDNA was loaded on an Agilent DNA chip (DNA-1000) to verify cDNA quality and quantity. Only libraries reaching satisfactory conditions were used for sequencing, on one sequencing lane of an Illumina Genome Analyzer II system (GAII, Illumina). The raw data generated during the sequencing procedure on the GAII will be deposited in Gene Expression Omnibus (GEO) under submission number GSE35296.

### RNA–seq data analysis

Sequencing reads were mapped to the human genome (version GRCh37/hg19) using the program gem-mapper from the GEM suite (http://gemlibrary.sourceforge.net). The GEM mapper reports exhaustively all mappings and split-mappings up to a user-defined amount of mismatches (default 2 mismatches), disregarding presumptive base-calling errors as identified by low associated quality values. Mapped reads were used to quantify transcripts from the RefSeq reference database [36], using the Flux Capacitor approach that deconvolves reads mapping to exonic regions shared by multiple transcripts by optimizing a system of linear equations and thus obtains a number of reads specifically assigned to each alternative spliceform (http://flux.sammeth.net, see [37] for a short description). All genes and transcripts have been assigned a relative coverage rate as measured in RPKM units (“reads per kilobase per million mapped reads”) [38].

Lists of differentially expressed genes and transcripts were generated from the Flux Capacitor output using scripts in Perl or R (see legends to figures and tables).

To define genes up- or downregulated by cytokines, the log_2_ of the proportion between the sum of the RPKM for all gene transcripts under cytokine condition and the same sum in control condition was taken as measure of change in gene expression. The p-value was obtained by performing a Fisher exact test (number of reads mapped to the gene and number of reads mapped to all other genes in the cytokine condition versus the control condition) and corrected by the Benjamini-Hochberg method (taking for each gene the 5 samples as independent tests). A difference in gene expression was considered significant if the corrected p-value was <0.05. As additional criteria, a gene was considered to be “modified by cytokines” only if its expression changed significantly in one direction - i.e. “up” or “down” - across at least 4 out of 5 islet preparations and no significant change in the opposite direction was observed. In order to quantify cytokine-modified splicing, differences in so-called “splice indices” - the proportion between the RPKM for a transcript and the sum of the RPKM for all the transcripts from the same gene - under cytokine exposure were compared to the control condition. Additionally, a p-value on the significance of changes in splicing patterns was obtained by performing a Fisher exact test (number of reads assigned to a certain transcript after deconvolution versus the number of reads mapped to all other transcripts of the same gene, comparing cytokine with control condition) and was corrected by the Benjamini-Hochberg method (taking for each transcript the 5 samples as independent tests). A change in AS was considered significant if the corrected p-value was <0.05. Consistent with the study of altered gene expression, a transcript was considered as “modified by cytokines” only if its splicing changed significantly in one direction - “up” or “down” - in at least 4 out of 5 islet samples and no sample pair exhibited a significant change in the opposite direction.

Preferential association of the lists of up/downregulated genes/transcripts with molecular and cellular functions and canonical pathways was determined with Benjamini-Hochberg corrected Fisher tests using the Ingenuity Pathway Analysis (IPA, Ingenuity Systems, http://www.ingenuity.com) software. A similar analysis was performed using DAVID (Database for Annotation, Visualization and Integrated Discovery, http://david.abcc.ncifcrf.gov) [39]. While IPA is curated manually, DAVID is generated automatically from 3^rd^ party databases. We used Gene Ontology Biological Process and Molecular Function, KEGG, InterPro and UCSC_TFBS for our DAVID analyses.

Networks of pairwise interactions between proteins, as described in the IntAct database, were obtained from the lists of up/downregulated genes using the PPI_spider [40] from the BioProfiling site (http://www.bioprofiling.de).

We employed an approach similar to the one used to define cytokine-modified genes to compare the untreated control islets to the adipose tissue, colon, kidney, liver and skeletal muscle tissue data available through the Illumina bodyMap2 project (accession number ERP000546 in the European Nucleotide Archive http://www.ebi.ac.uk/ena/data/view/ERP000546). A more detailed comparative analysis between pancreatic islets and other tissues, aiming to detect novel beta cell biomarkers, is under way and will be the subject of a future publication. The RPKM data and lists of cytokine-modified and human islet-specific genes are available in Dataset S1.

### Human islet and rat beta cell RNA extraction, RT–PCR, and qRT–PCR

Human islet preparations for validation experiments were from donors other than those used for sequencing (Table 1). In some experiments confirmation was also done in clonal INS-1E and primary rat beta cells, to confirm that gene expression was indeed derived from beta cells (human islets contain different cell types, with beta cells constituting around 60% of the total population in the present samples; Table 1). Poly(A)+ mRNA was isolated using the Dynabeads mRNA DIRECT kit (Invitrogen, Paisley, UK) and reverse transcribed as previously described [26]. Quantitative PCR was performed using the iQ SYBR Green Supermix (BIO-RAD, Nazareth Eke, Belgium) on a LightCycler (Roche Diagnostics, Mannheim, Germany) or iCycler MyiQ Single Color (BIO-RAD) instrument [41], [42]. Data were expressed as number of copies using the standard curve method. Expression values were corrected for the housekeeping gene β-actin and/or glyceraldehyde-3-phosphate dehydrogenase (GAPDH). These housekeeping genes are not modified by pro-inflammatory stimuli under the present experimental conditions [43], [44], [45]. For the evaluation of splice variant expression, conventional PCR was done. Primers were designed for DnaJ homolog subfamily A member 3 (DNAJA3) on exon-spliced junctions between exon 9 and 11 to obtain a product of 267 bp for variant 1 (NM_005147.4) and 150 bp for variant 2 (NM_001135110.1). RNA from INS-1E cells, transfected with a control siRNA (siC) or a siRNA targeting Nova1, was retro-transcribed and the cDNAs amplified with gabrg2 primers. The samples were amplified using BioTAQ Red DNA Polymerase, 10× NH_4_ reaction buffer, 50 mM MgCl_2_ and 100 mM dNTP mix (BioLine, London, UK) in a Thermal Cycler (Applied Biosystems) using the following conditions: after 8 min of denaturation at 95°C, samples were run for 32–35 cycles consisting of 1 min at 95°C, 45 sec at 60°C and 1 min at 72°C. The final step was 5 min at 72°C. PCR products were visualized on 2.3% agarose gel, stained with SYBR Safe gel stain (Invitrogen). Primers used for qRT- and RT-PCR are listed in Table S1.

### RNA interference

For RNA interference in rat beta cells, the following siRNAs were used: smart pool targeting MDA5 (reference 105259, Thermo Scientific), siBCL2A1 CAGGGAAGAUCUGGGAAAUGCUCUU, smart pool targeting BCL2A1 (reference 170929, Thermo Scientific), siNova1 stealth UUAGCAUGUCCUAAUAGCCCUGCGG (Invitrogen) and Allstars Negative Control siRNA (Qiagen, Venlo, the Netherlands). Cells were transfected with a mix of 30 nM of siRNA and Lipofectamine RNAiMAX (Invitrogen) diluted in Opti-MEM I (Invitrogen) as described [5]. The transfection efficiency was >90% [5], [46]. After overnight transfection the cells were cultured for 48 h before being retrieved for evaluation of RNA and protein expression.

### Western blot and chemokine and cytokine ELISA

For Western blotting, equal amounts of proteins were loaded in 12% SDS-PAGE. Immunoblot analysis was performed using goat anti-Nova1 (0.03 µg/ml; Abcam, Cambridge, UK) and mouse anti-α-tubulin (1∶5000; Sigma) antibodies. The proteins were detected using horseradish peroxidase-conjugated secondary antibody (1∶5000; Santa Cruz Biotechnology) and chemiluminescence Supersignal (Pierce). Densitometric analysis was performed using analysis software Aida1D (Fujifilm, London, UK) and data were normalized for α-tubulin.

Release of the human chemokines CXCL1 (Gro-α), CXCL9 (Mig), CXCL10 (IP-10), CXCL11 (Itac), CCL2 (MCP-1), CCL3 (Mip-1-α), CCL5 (Rantes) and the cytokines IL-6 and IL-8 was measured in culture medium of control and cytokine-exposed human islets using a Custom Multi-Analyte ELISArray kit (SABiosciences, Frederick, MD, USA). Samples were processed following the manufacturer's instructions. This is a semi-quantitative assay that does not include a standard curve. Absorbance at 450 nm was measured, corrected by readings at 570 nm, normalized to the geometric mean of β-actin and GAPDH expression and expressed as arbitrary units.

### Immunofluorescence

Human pancreatic tissue obtained from biopsies or organ donors were fixed in formaldehyde and embedded in paraffin. Sections were stained for double immunofluorescence with rabbit anti-Nova1 (1∶500; Merck-Millipore, Overijse, Belgium) and guinea pig anti-insulin (I2018, 1∶2000; Sigma) or mouse anti-glucagon antibodies using FITC and Cy3 as fluorochromes, respectively. The samples were analyzed by inverted fluorescence microscopy and images captured with Axiocam (Zeiss).

### Assessment of apoptosis

The percentage of apoptotic cells was determined by two observers (one being blind to sample identity), after staining with the DNA-binding dyes propidium iodide and Hoechst 33342 (Sigma-Aldrich) as previously described [47]. At least 500 cells were counted per condition, with an agreement between findings obtained by the two observers of >90%.

### Statistical analysis

Data for the confirmation experiments are presented as means ± SEM. Comparisons were performed by paired two-tailed Student's t-test or Mann Whitney test as indicated in the figure legends. A p-value≤0.05 was considered statistically significant. The statistical analysis of the RNA-seq data is described above.

## Results

### Sequencing of human islets and analysis of transcripts

RNA-seq data were obtained from 5 human islet preparations (Table 1) cultured under control condition or following a 48-h exposure to the cytokines IL-1β+IFN-γ. Each of these preparations was sequenced on a single lane of an Illumina GAII sequencer, with 10–51 million reads for control and 35–62 million reads for cytokine-treated islets. This provides sufficient sequencing depth to quantify gene expression and detect rare transcripts as previously shown [16]. The 51 nucleotide paired-end reads were mapped to the human genome (version hg19) using GEM software. Taking this approach, we were able to map on average 83% of the raw reads. GEM can report multiple mappings for a single read and we observed on average a redundancy (mappings to reads ratio) of 1.5 (Table S2).

Reads that align with exons or with overlapping exon junctions can be used to evaluate the levels of splicing. We used the Flux Capacitor software, which in brief takes as input a list of reads mapped to the genome and a list of transcript annotations, and subsequently produces a list of reads that are uniquely assigned to one of the transcripts. As reference transcript annotation, we employed the 34,102 annotated human mRNA and ncRNA sequences from RefSeq [48]. In a first step, the program interprets the mate information of mappings and filters off mappings that do not pair properly within the boundaries of annotated transcripts. For about half of the originally sequenced reads a mate in correct orientation and within exon boundaries of the annotated RefSeq transcripts could be identified, with only spurious redundancy (<1.01). The 34,102 transcripts from RefSeq correspond to 22,205 genes, and islets cultured under control condition were found to express a median of 17,787 genes, with numbers varying with sequencing depth (Table S2). Of these, 15,212 genes were expressed in all individuals while 3,841 genes were expressed in some but not all. 5,408 genes expressed in all individuals have AS annotated in RefSeq (see below).

**Figure 1:**
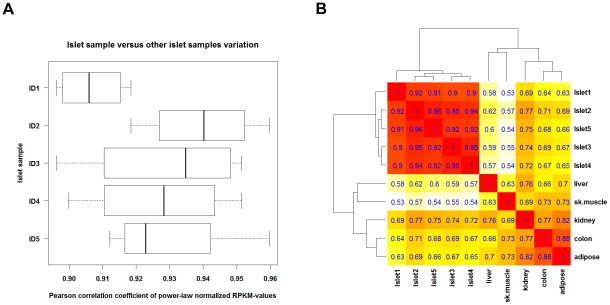
Correlation between RNA-seq gene expression levels. Gene expression levels were compared among the 5 islet preparations (cultured under control condition) and between islets and 5 selected background tissues from the Illumina Human Body Map. Only genes with an RPKM>1 in all samples were considered for analysis. For each pair of samples a Pearson correlation coefficient (PCC) was computed from the power-law normalized expression levels (i.e. the RPKM values). (A) Boxplot for each islet sample (called ID1 to ID5) with the PCC values between the individual islet sample and 4 other islet preparations. (B) Heatmap with clustering dendrograms inferred by employing (1 – PCC) as distance function and complete linkage as clustering function, showing a tight cluster of islet preparations.

Analyses of the qualitative agreement of expression levels between the individual islet preparations using Pearson correlation coefficients (PCC) indicated a high correlation (0.95) (Figure 1A). As gene expression follows Zipf's law [49], corresponding quantification values have been power-law normalized to meet the prerequisite for correlation studies [50]. For each sample-pair, the corresponding PCC provides a numerical condensation of the similarity between gene expression profiles: a PCC of 1.0 represents sample-pairs where all expression tuples fall along a line, whereas a PCC of 0 is assigned to sample pairs that do not exhibit linear correlation. For the purpose of comparison, 5 tissues from the Illumina Human Body Map project, i.e. colon, adipose, kidney, liver and skeletal muscle, have been subjected to an analogous procedure. The similarity in terms of correlation among gene expression levels was significantly higher between the islet samples (0.90–0.96) than in comparison with the 5 other tissues (0.53–0.88). In line with these observations, a heatmap with complete linkage as clustering function indicates that the 5 islet preparations clustered together, as compared to the other tissues (Figure 1B). It cannot be excluded that islet culture affects the human islet transcriptome, although in other studies differential gene expression between diabetic and non-diabetic individuals was maintained after culture [25], [51], [52].

For internal methodological validation, we selected 4 genes for confirmation by qRT-PCR in the same samples used for RNA-seq. The gene expression data using these two methods were essentially superposable (Figure S1).

The validation steps described above, including comparison between islet samples and against 5 other tissues, and the validation using qRT-PCR in the same samples, indicate that the RNA-seq of human islets provided reliable and reproducible data, as has been described for other tissues [11], [16], [38], [53], enabling us to proceed with the analyses described below.

### Expression of candidate genes for type 1 diabetes in pancreatic islets

**Figure 2:**
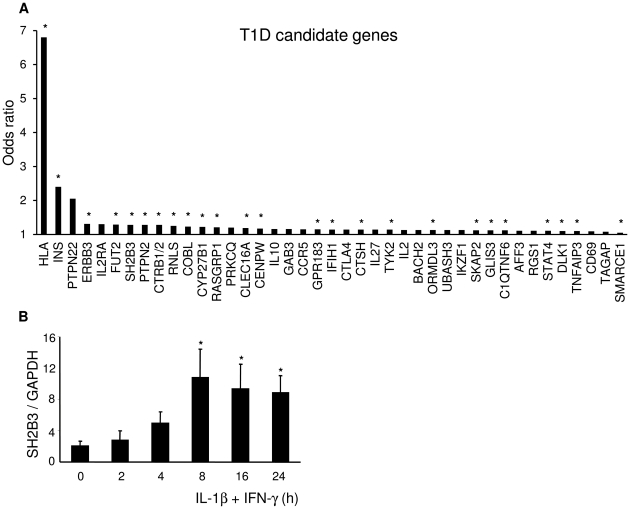
Two thirds of candidate genes for T1D are expressed in pancreatic beta cells. (A) T1D candidate genes ranked by the odds ratio for their risk allele (http://t1dbase.org). Based on our present data, 25 candidate genes out of 41 (61%) were expressed in human beta cells (marked with *). (B) INS-1E cells were left untreated or treated with IL-1β+IFN-γ for the indicated times. The expression of the T1D candidate gene *SH2B3* was assayed by qRT-PCR and normalized to the housekeeping gene *GAPDH*. The results are means ± SEM of 3–6 independent experiments. *p<0.05 versus untreated cells.

Based on the datasets above, we examined whether candidate genes for T1D, previously identified by genome-wide association studies (GWAS) [54], [55], are expressed in human islets. We considered genes as “expressed” with a median RPKM >1. Out of 41 candidate genes, 25 (i.e. 61%) were clearly expressed in human islets (Figure 2A and Table S3). We followed this up by functional studies in insulin-producing INS-1E cells and purified rat beta cells, to confirm gene expression and query the relevance of these genes at the beta cell level. We have previously shown that 2 of these genes, namely *IFIH1*/*MDA5* and *PTPN2*, are expressed in pancreatic beta cells and regulate respectively local inflammation [6] and apoptosis [5], [56]. Pro-inflammatory cytokines and dsRNA, a by-product of viral infections, modulate expression of these 2 genes, indicating crosstalk between T1D candidate genes and environmental factors and local inflammation [5], [6], [56]. Indeed, knockdown of IFIH1/MDA5 in rat beta cells reduced the chemokine and cytokine expression induced by a 48-h exposure to PIC, a synthetic dsRNA (Figure S2). We now confirm in clonal INS-1E cells expression of an additional candidate gene, namely *SH2B3* (Figure 2B), and its induction by the cytokines IL-1β+IFN-γ in a time-course study.

It is commonly thought that antioxidative defense mechanisms of pancreatic beta cells are very low, rendering the cells vulnerable to reactive oxygen species which contribute to the pathogenesis of diabetes (reviewed in [57]). This seems to be the case for rat beta cells [58], but we have previously shown that human beta cells are 5–10-fold more resistant than mouse or rat islets to oxidative stress generated by agents such as alloxan [33]. We compared expression of several free radical scavenging enzymes in human islets against 5 other tissues (Table S4). Human islets have robust expression of several of these enzymes, including a marked expression of catalase (median RPKM 26) and *SOD2* (median RPKM 388). In line with these findings, we have previously observed that human islets have several-fold higher expression of antioxidant enzymes than rodent islets [59]. Expression levels in islets compared to liver were lower for 3 antioxidant enzymes, similar for 3 and significantly higher for 4 enzymes, suggesting that human islets, as opposed to rodent islets, may have a fair antioxidant capacity.

### Analysis of cytokine-modified genes

**Figure 3:**
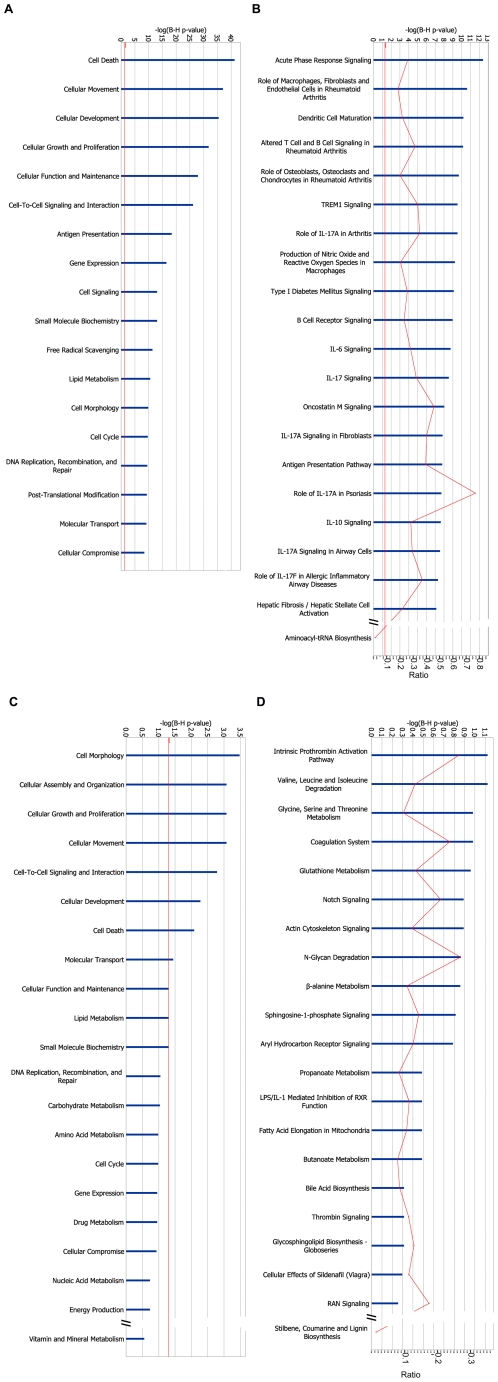
IPA of cytokine-modified genes. (A, B) 1,416 genes were significantly up-regulated by the cytokines IL-1β+IFN-γ in at least 4 out of 5 islet samples, and significantly downregulated in none. These genes were mapped to 1,398 unique entries in the IPA database, which were submitted to gene set enrichment analysis based on Benjamini-Hochberg corrected Fisher tests. IPA of these cytokine-up-regulated genes is shown for (A) “Molecular and Cellular Function” and (B) “Canonical Pathways”. (C, D) 1,652 genes were significantly downregulated by cytokines in at least 4 out of 5 islet samples, and significantly up-regulated in none. They were mapped to 1613 unique entries in the IPA database. IPA of these cytokine-downregulated genes is shown for (C) “Molecular and Cellular Function” and (D) “Canonical Pathways”. The length of the blue bars indicates the significance of the association between the set of genes and the keyword, and is expressed as minus the logarithm of the probability that a random set of genes from the human genome would be associated with the same keyword. The straight red line indicates a threshold of 0.05 (corresponding to a −log(B–H p-value) of 1.3). The curved red line indicates for each pathway the ratio between the number of genes observed in the data set and the total number of genes in the pathway (as annotated in IPA).

From the 19,621 genes detected as “present” by the RNA-seq, a total of 3068 (16%) were significantly modified by a 48-h exposure to the pro-inflammatory cytokines IL-1β+IFN-γ. From these, 1416 and 1652 were respectively up- and downregulated. The complete list of cytokine-modulated genes is accessible at http://lmedex.ulb.ac.be/data.php; password will be provided upon request. These genes were manually curated (by DLE; see selected cytokine-modified genes in Table S5) or analyzed in a non-biased way using IPA (Figure 3). Table S5 indicates that many key beta cell functions were modified by cytokines, including glucose and lipid metabolism, protein synthesis and translation, kinases and phosphatases and transcription factors. The most important responses, however, were those related to inflammation, innate immune response and apoptosis. Thus, there was massive up-regulation of the expression of a large number of genes encoding chemokines and cytokines, of genes involved in IFN-γ signaling and NF-κB regulation, proteasome/antigen presentation, and other innate immune response/pro-inflammatory components. There was also up-regulation of many genes involved in apoptosis, free radical scavenging and DNA damage response (Table S5). These observations were supported by IPA, which showed that up-regulated genes belong prominently to the functions “Cell Death” and “Cellular Movement” (actually mainly chemokines) (Figure 3A). In the IPA “Diseases and Disorders” analysis (not shown) modified genes clustered in “Inflammatory Response”. As shown in Figure 3B, IPA canonical pathways indicated that the highest p-value was related to “Acute Phase Response Signaling”. Interestingly, among the top canonical pathways we also found several other inflammation-related headlines, such as “Role of macrophages…”, “Dendritic cell…”, “Altered T and B cell signaling”, “IL-17 signaling” and, reassuringly, “Type 1 diabetes mellitus signaling”. The fact that 6 of the top canonical pathways were related to IL-17 is of particular interest given that IL-17 signaling may play a direct role in beta cell apoptosis in human T1D [43].

**Figure 4:**
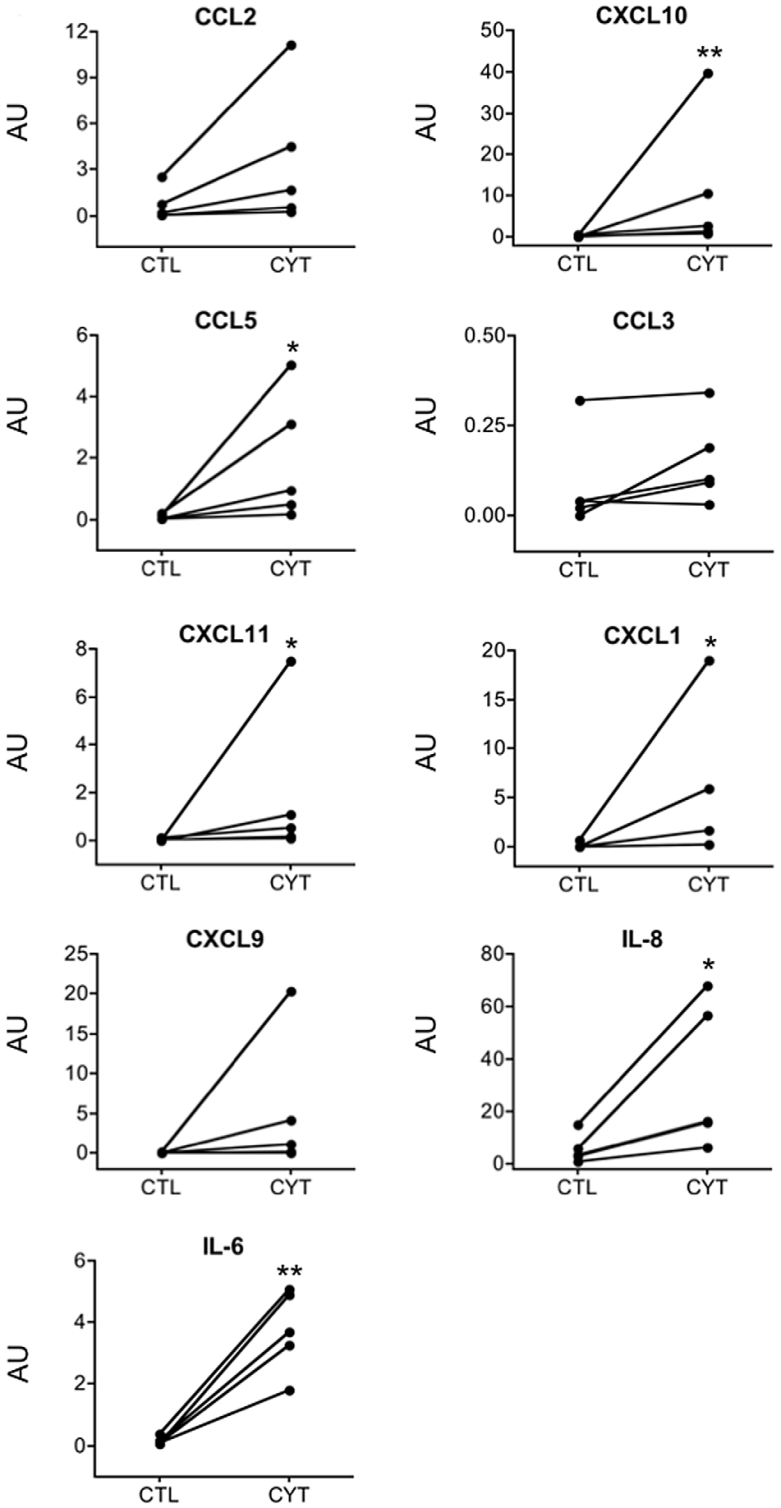
IL-1β+IFN-γ induce chemokine and cytokine protein expression in human islets. Human islets from 5 organ donors were cultured for 48 h in the presence (CYT) or absence (CTL) of cytokines. Chemokines and cytokines secreted to the culture medium were measured by ELISA. Data were normalized to the geometric mean of *β-actin* and *GAPDH* expression and expressed as arbitrary units (AU). *p<0.05, **p<0.01 for CYT vs CTL by Mann Whitney test.

The analysis using IPA was validated by a separate analysis using the public tool DAVID, which relies on copies of various public databases. A term enrichment analysis against Gene Ontology, KEGG (metabolic and regulatory pathways) and InterPro (protein conserved motifs) showed that the up-regulated genes were preferentially associated with immune response, apoptosis, cytokines and other terms related to inflammatory stress (Figure S3A–S3D). Noteworthy is that term enrichment analysis against UCSC_TFBS showed genes with potential binding sites for the transcription factors NF-κB, AP-1 (Jun) and BACH2 (not shown). Protein-protein interactions among the up-regulated genes were examined using the BioProfiling tool, which relies on the IntAct database (Figure S4). It shows several interactions related to inflammatory response and antigen processing and presentation, with a clear role for members of the NF-κB and STAT families. The observations by RNA-seq of cytokine-induced chemokines (Table S5) are in line with our previous observations using array analysis of human islets exposed to viral infection or pro-inflammatory cytokines [60] or qRT-PCR of human and mouse islets exposed to cytokines or isolated from pre-diabetic mice [42]. The RNA-seq findings were confirmed at the protein level by ELISA for nearly all chemokines studied (Figure 4), indicating that many of the observed gene expression changes are translated to functional proteins with potential relevance for the early pathogenesis of T1D.

“Molecular and Cellular Function” IPA showed that downregulated genes belonged to “Cell Morphology”, “Assembly and Organization”, “Growth and Proliferation” and “Movement” (Figure 3C) and amino acid metabolism in the IPA “Canonical Pathways” (Figure 3D). A DAVID term enrichment analysis produced similar results (Figure S3E–S3H).

We compared the presently observed cytokine-modified genes in human islets against our recently published array data in cytokine-exposed INS-1E cells [61]. For this comparison, we used genes with homology between human and rat, and probes present on the Affymetrix GeneChip Rat Genome 230 2.0 array. This selection encompassed 790 and 874 genes, considered as up- or down-regulated by RNA-seq, respectively. Of these, 53% and 50% were detected as respectively “up-” and “down-regulated” in cytokine-treated INS-1E cells (data not shown). When we focused on some of the most relevant cytokine-modulated genes (Table S5) the overlap was even higher between human islet and INS-1E genes, with respectively 76% and 63% of the NF-κB/other transcription factors and chemokines showing a similar variation. Considering the issues of species differences (human vs rat), differences between primary/clonal cells (islets vs INS-1E cells), methodological differences (RNA-seq vs array analysis to asses RNA expression) and timing of exposure to cytokines (48 h for human islets and 12–24 h for INS-1E cells), the observed correlation (50–53%) between genes expressed in human islets and INS-1E cells is reasonable, and suggest that many of the presently observed cytokine-modified genes are expressed in beta cells.

**Figure 5:**
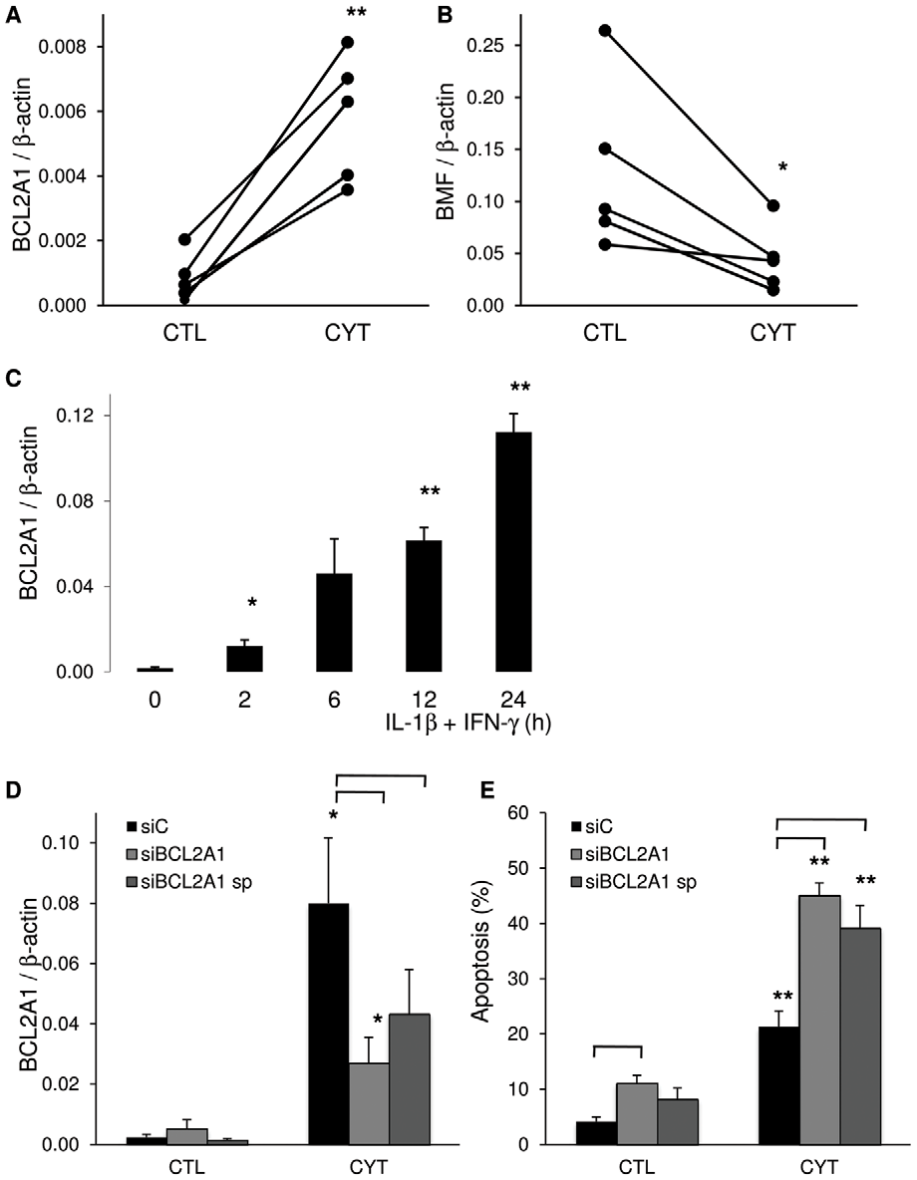
IL-1β+IFN-γ modifies BCL2A1 and BMF expression. (A, B) RNA-seq data were validated for *BCL2A1* and *BMF* by qRT-PCR in 5 independent human islet preparations exposed or not (CTL) to the cytokines IL-1β+IFN-γ (CYT). Data were normalized to expression levels of the housekeeping gene *β-actin*. (C) INS-1E cells were left untreated or treated with IL-1β+IFN-γ for different times. *BCL2A1* expression was assayed by qRT-PCR and normalized for *β-actin* expression. The results are means ± SEM of 4 independent experiments. (D, E) INS-1E cells were transfected with control siRNA (siC, black bars) or either a single or a combination of 4 siRNAs (smart pool, sp) targeting *BCL2A1* (siBCL2A1, grey bars). After 48 h, cells were exposed to IL-1β+IFN-γ (CYT) for 16 h. (D) *BCL2A1* expression was measured by qRT-PCR and normalized for *β-actin* expression. Results are mean ± SEM of 4 independent experiments. (E) Apoptosis was examined by fluorescence microscopy after staining with the DNA-binding dyes propidium iodide and Hoechst 33342. Results are mean ± SEM of 4 independent experiments. *p<0.05, **p<0.01 versus untreated control (CTL); p<0.05 for the comparison siC versus siBCL2A1 as indicated.

To further confirm expression of some of the cytokine-modified genes, we used independent samples for qRT-PCR evaluation. We selected genes potentially involved in apoptosis, namely Bcl-2 related protein A1 (*BCL2A1*) and Bcl-2 modifying factor (*BMF*) (Figure 5). In line with the RNA-seq data (Table S5), cytokines respectively increased and decreased expression of *BCL2A1* and *BMF* (Figure 5A and 5B). In clonal INS-1E cells, *BCL2A1* expression was induced by IL-1β+IFN-γ in a time-course study (Figure 5C). Efficient knockdown of BCL2A1 using two different siRNAs (Figure 5D) amplified cytokine-induced apoptosis (Figure 5E), demonstrating the anti-apoptotic role of BCL2A1 in beta cells. Another Bcl-2 family member that was up-regulated by cytokines in the RNA-seq is *BBC3* (*PUMA*, Table S5). BBC3 was recently shown to be cytokine-induced at the mRNA and protein level and pro-apoptotic in beta cells [23].

Of interest, expression of several of the putative candidate genes for T1D (Figure 2) was modified by 48-h exposure to cytokines. Besides the ones already discussed above (*PTPN2*, *IFIH1* and *SH2B3*), *STAT-4*, *GLIS-3*, *CD55*, *RASGRP1*, *SKAP2* and a large number of HLA-related genes tended to increase expression following cytokine exposure (Table S5).

### Splice variants in human islets and their regulation by Nova1 and pro-inflammatory cytokines

**Figure 6:**
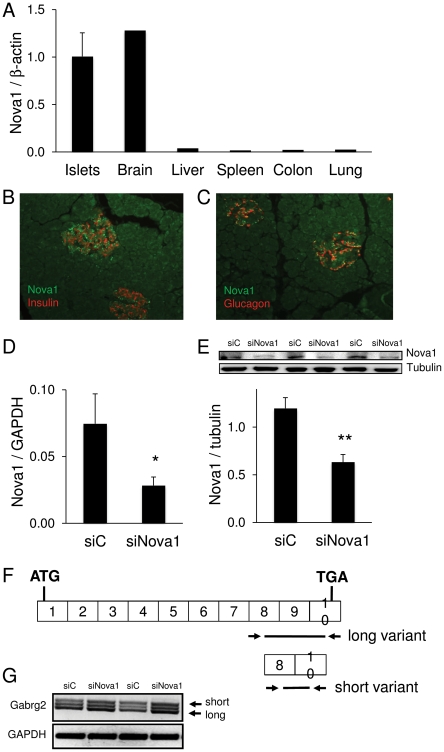
Nova1 expression and function in human pancreatic islets. (A) *Nova1* mRNA expression was examined by qRT-PCR in 7 human islet preparations and human brain, liver, spleen, colon and lung tissue. Data were normalized to expression levels of the housekeeping gene *β-actin*. (B, C) Nova1 expression (green) was evaluated by immunofluorescence in human pancreatic sections stained for insulin (B) or glucagon (C, either hormone labeled red). The picture is representative of 3 independent experiments. (D–G) Splicing by Nova1 was examined in INS-1E cells transfected with control (siC) or Nova1 siRNA (siNova1). Efficient Nova1 knockdown was shown by qRT-PCR (D) and Western blot (E) (n = 3). (F) To evaluate the splicing function of Nova1, RT-PCR was performed in siC and siNova1 transfected INS-1E cells, using primers flanking exon 9 of *Gabrg2*. (G) Nova1 knockdown, expected to lead to less exon 9 inclusion, increased the abundance of the short Gabrg2 transcript variant. The picture is representative of 3 independent experiments. *p<0.05, **p<0.01.

Within the 5 islet samples, we found evidence for 87.3% of the islet-expressed genes that have multiple RefSeq transcripts annotated to express more than one spliceform. The complete list of these transcripts is available online at http://lmedex.ulb.ac.be/data.php; password will be provided upon request. Since there is no available information on the regulation of splicing in human or rat islet cells, we examined the expression in human islets of 224 genes previously identified as splicing factors in other human tissues [62] and found that most of them are expressed in islets, and 69 significantly more than in at least 4 out of 5 selected background tissues (adipose tissue, colon, kidney, liver and skeletal muscle, data not shown). We detected expression of several so-called “neuron-specific” splicing factors, including *Nova1*. Nova1 participates in the splicing of several genes implicated in neuronal function and development [63], [64], and was previously detected by microarray profiling of human islets [65]. We confirmed by qRT-PCR that Nova1 is indeed well expressed in human islets, at levels comparable to brain and higher than in liver, spleen, colon and lung (Figure 6A). Expression of Nova1 at the protein level was confirmed in insulin-positive beta and glucagon-positive alpha cells in human pancreatic sections, while there was little or no staining in the exocrine pancreas (Figure 6B and 6C). To explore the splicing function of Nova1 in beta cells, the gene was knocked down by a specific siRNA in insulin-producing INS-1E cells, leading to a nearly 60% decrease in Nova1 mRNA and protein expression (Figure 6D and 6E). To test the functional impact of Nova1 knockdown, we evaluated the expression of splice variants of gamma-aminobutyric acid A receptor, gamma 2 (Gabrg2). Nova1 was previously shown to cause exon 9 inclusion in Gabrg2 transcripts in mouse brain [66]. Primers were designed on the flanking regions of this exon (Figure 6F) to differentiate between the long transcript variant with exon 9, the short variant without exon 9 and an intermediate undefined variant [67]. Knockdown of Nova1 modified the splicing pattern of the gabrg2 transcripts generating more of the short variant (Figure 6G), suggesting a functional role for this splicing factor in beta cells.

**Figure 7:**
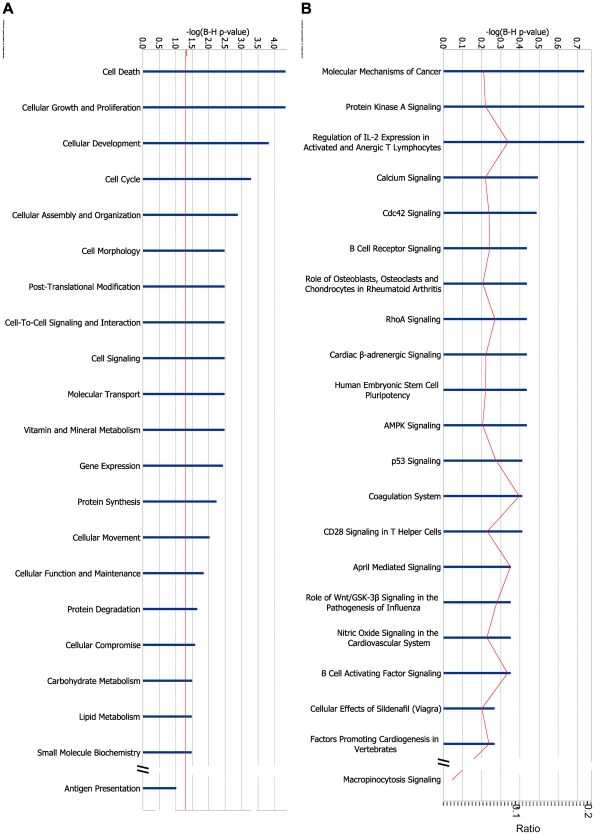
IPA of changes in alternative splicing induced by cytokines. IPA of genes with AS modified by IL-1β+IFN-γ. 425 transcripts were significantly up-regulated in at least 4 out of 5 islet samples and significantly downregulated in none, and 433 transcripts were significantly downregulated using similar criteria. These transcripts could be mapped by RefSeq ID to 546 genes. IPA of these genes for (A) “Molecular and Cellular Function” and (B) “Canonical Pathways”. The length of the blue bars indicates the significance of the association between the set of transcripts and the keyword, and is expressed as minus the logarithm of the probability that a random set of transcripts from the human genome would be associated with the same keyword. The straight red line indicates a threshold of 0.05 (corresponding to a −log(B–H p-value) of 1.3). The curved red line indicates for each pathway the ratio between the number of transcripts observed in the data set and the total number of transcripts in the pathway (as annotated in IPA).

Exposure of human islets to the cytokines IL-1β+IFN-γ induced modifications in the splicing of 548 genes; of these 425 and 433 splice variants were respectively up- and downregulated by the cytokines, as evaluated by a conservative assessment (see Methods). IPA of transcripts exhibiting cytokine-modified AS indicates that a large number of transcripts were related to “Cell Death” or “Cellular Growth and Proliferation” (Figure 7A) and canonical pathways of T and B cells and PKA, calcium, AMPK and p53 signaling (Figure 7B). A DAVID term enrichment analysis yielded among the top terms “cell death” and “apoptosis” (not shown).

To validate the RNA-seq analysis, we selected DNAJA3 for PCR confirmation in independent samples. DNAJA3 is related to “Cell Death” and its variants 1 and 2 were respectively down- and up-regulated by cytokines in 5 out of 5 islet samples. By RT-PCR, the cytokines IL-1β and IFN-γ increased variant 2 expression in 3 independent human islet preparations (Figure S5).

## Discussion

We presently describe the first global sequencing of RNAs expressed in human islets of Langerhans. The analysis identified 15,200 genes expressed in the five independent preparations, increasing by >2-fold the known expressed genes in human islets. There was a high correlation between the islet samples (0.90–0.96), clearly higher than the correlation observed between islets and five other tissues (0.53–0.88) used for external comparison. This, and the fact that around 20 genes identified as expressed and/or modified by cytokines in the present analysis were confirmed at the RNA and/or protein expression level by other methods, supports the reliability of the present observations. This is in line with previous studies in other tissues indicating that RNA-seq is a reliable and reproducible method to evaluate RNA expression [11], [16], [38], [53].

The human islets used in this analysis contained 58% beta cells on average (Table 1), and the transcriptome includes RNAs from non-beta endocrine cells, mostly alpha and delta cells [51], and ductal cells. The comparison against INS-1E cells suggests, nonetheless, that at least half of the presently identified cytokine-modified genes are expressed in beta cells.

Use of GWAS has revealed more than 40 loci containing putative genetic contributors to the pathogenesis of T1D [54], [55]; this number was further increased by a recent genome-wide meta-analysis of six diabetes cohorts [68]. While in T2D most candidate genes impact more on islet function than on insulin resistance and are hence considered to regulate beta cell function and development [69], [70], it is usually assumed that in T1D most if not all candidate genes modulate the immune system (reviewed in [21]). In this conventional view beta cells are regarded as “passive victims” of a process that starts and is regulated elsewhere. By using the presently generated datasets, we observed that 61% of the candidate genes for T1D are consistently expressed in human pancreatic islets. Furthermore, the present and previous observations [5], [6], [56] indicate that expression of many of these genes change following exposure to pro-inflammatory cytokines or dsRNA (a by-product of virus infection), agents that may contribute to triggering of T1D [2]. For at least two of these genes, namely *IFIH1*/*MDA5* [6] (present data) and *PTPN2* [5], [6], [56], there is experimental evidence pointing to their respective roles in production of chemokines/cytokines and beta cell apoptosis.

These observations are in line with the present analysis of gene expression in cytokine-treated human islets. Of note, only one time point (48 h cytokine exposure) was examined here, providing a snapshot of dynamic regulation of gene expression. It is conceivable that relevant cytokine-modulated genes at other time points were missed in the present analysis. Cytokines modified expression of 3,000 genes, mostly related to inflammation, innate immune response and apoptosis. Key chemokines and cytokines were among the most up-regulated genes in human islets, a finding confirmed at the protein level for *CCL2*, *CCL5*, *CCL3*, *CXCL9*, *CXCL10*, *CXCL11*, *IL-6* and *IL-8*. This is in good agreement with findings in diabetes-prone NOD mice, where increased expression of CCL2, CXCL10 and other chemokines/cytokines are observed in the pre-diabetic period [42], [71], [72]. CCL2 and CXCL10 attract macrophages, and may contribute to the recruitment of immune cells during the early stages of insulitis, as suggested by the observation that transgenic expression of CCL2 in beta cells causes insulitis and diabetes [72]. Some of these observations have been recently confirmed in histological material from T1D patients. Thus, it was observed that pancreatic beta cells from islets affected by insulitis express *CXCL10*, while the infiltrating T cells express *CXCR3*, the receptor of CXCL10 [73], [74]. Islet cells themselves are probably an important source of chemokine production during inflammation, as suggested by the present findings. That chemokines are indeed produced by beta cells is supported by the observations that FACS-purified rat beta cells (>90% pure) or clonal rat beta cells (INS-1E cells) exposed to IL-1β+IFN-γ, or to dsRNA, show increased expression of mRNAs encoding *CCL2*, *CXCL10*, *CCL20*, *CX3CL1* and *IL-15*, among others [9], [44], [61], [75]. This is confirmed by histology of pancreatic samples, showing expression of chemokines by beta cells [73], [74], [76].

The findings described above support the concept of a “dialogue” between beta cells and the invading macrophages and T cells in the course of insulitis, rather than a “monolog” where all action takes place at the level of the immune system and beta cells are no more than passive victims. Thus, activated mononuclear cells produce cytokines such as IFN-γ, IL-1β and TNF-α, triggering the release of chemokines and stimulatory cytokines by the beta cells. This, together with beta cell death and the putative presentation of neoantigens secondary to modified AS and up-regulation of the machinery for antigen presentation, will attract more mononuclear cells that also release multiple cytokines and chemokines, in a process modulated by candidate genes that are expressed and act at both the immune system and beta cell levels, as shown for *MDA5* and *PTPN2*, among others.

One of the most deleterious consequences of islet inflammation is the progressive loss of pancreatic beta cells via apoptosis [2]. We presently observed modulation of the expression of several apoptosis-related genes in human islets exposed to cytokines. One of them, the anti-apoptotic Bcl-2 family member *BCL2A1* [77], [78], was confirmed by qRT-PCR in both independent human islet preparations and in clonal rat insulin-producing INS-1E cells. Knock down of BCL2A1 by a specific siRNA augmented both basal and cytokine-induced apoptosis, confirming the relevant function of this protein in protecting beta cells against apoptosis (present data). Cytokine-induced expression of *BCL2A1* in human islets has been previously observed by array analysis [60], [79], but the function of this gene in beta cells remained to be clarified. Of interest, BCL2A1 inhibits apoptosis induced by, among others, the BH3 only protein Bim [80], [81]. Bim was recently shown to be a crucial pro-apoptotic signal following inhibition of the candidate gene *PTPN2* [56], a gene also detected in the present RNAseq.

We presently report another level of molecular regulation of beta cell function, namely AS. Interestingly, AS is modified by cytokine exposure as suggested by the present findings in human islets and previous observations from our group based on exon array analysis in rat beta cells [9]. Regulation of splicing in other tissues involves the cooperation between SR, hnRNPs proteins and several other tissue-specific regulators of splicing such as neuron-specific Nova or the neural/muscle-enriched Fox proteins [82], [83]. The well-characterized Nova proteins regulate numerous splicing events in the central nervous system [64], [84], and the present findings show that Nova1 is expressed in beta cells and affects splicing of at least one target gene, namely Gabrg2. Of interest, several of the known Nova target genes in brain are also expressed in beta cells, including neuroligin and neurexin family members, inhibitory synapse-associated neuroligin and neurexin binding partners [64], [85]. These findings are in line with previous observations that beta cells share expression of a large number of genes and proteins with the central nervous system [86], [87], [88]. This opens a new field of research, and new experiments are now required to determine how AS is regulated in beta cells, and how cytokines modify this process.

In conclusion, the present study identifies most of the transcripts present in human islets of Langerhans, providing a valuable dataset for future genetic and functional studies in pancreatic beta cells. It also shows that pro-inflammatory cytokines modify AS and the expression of nearly 20% of the genes expressed in human islet cells. Importantly, the present observations indicate that >60% of the known candidate genes for T1D are expressed in human islets. This, taken together with the cytokine-induced expression of a large number of chemokines and cytokines in human islets, reinforces the concept of a dialog between pancreatic islets and the immune system, which might be crucial for triggering insulitis and eventual progression to diabetes. The present study identifies a large number of the words used by pancreatic islets in this dialog, and points to candidate genes for T1D as one of the writers of the beta cell speeches.

## Supporting Information

Dataset S1 RPKM data and lists of cytokine-modified and human islet-specific genes. Includes “table_RPKM.xlsx” (detected transcripts with their expression levels), “ctrl_cyt_expr.up.xlsx” (list of upregulated genes), “ctrl_cyt_expr.down.xlsx” (list of downregulated genes), “ctrl_cyt_AS_up.xlsx” (list of upregulated splicing isoforms), “ctrl_cyt_AS_down.xlsx” (list of downregulated splicing isoforms) and “Legends.docx” (explanation of the tables).
(ZIP)

Figure S1 Validation of RNA-seq gene expression data by qRT-PCR in cytokine-treated human islets. Human islets from 5 organ donors were cultured for 48 h in the presence (CYT) or absence (CTL) of the cytokines IL-1β+IFN-γ. RNA-seq gene expression results (black bars) were compared to gene expression assessed by qRT-PCR (gray bars) in the 5 human islet preparations used for RNA-seq. Data were normalized to the geometric mean of *β-actin* and *GAPDH* expression and expressed as fold induction of control. *p<0.05, **p<0.01 for CYT versus CTL.
(TIF)

Figure S2 MDA5 regulates cytokine and chemokine production in primary rat beta cells exposed to intracellular dsRNA. FACS-purified rat beta cells were transfected with control siRNA (siC, black bars) or siRNA targeting MDA5 (siMDA5, grey bars). After 48 h, cells were left untreated or transfected with PIC for 48 h. *MDA5*, *IFN-β*, *CCL5* and *CXCL10* mRNA expression was assayed by qRT-PCR and corrected for the housekeeping gene *GAPDH*. Results are mean ± SEM of six independent experiments. *p<0.05, **p<0.01, ***p<0.001 versus control; p<0.05 for the comparison siC versus siMDA5 as indicated.
(TIF)

Figure S3 DAVID analysis of cytokine-modified genes. (A, B, C, D) 1,416 genes were significantly up-regulated by the cytokines IL-1β+IFN-γ in at least 4 out of 5 islet samples, and significantly downregulated in none. These genes mapped to 1,395 unique entries in the DAVID database, which were submitted to gene set enrichment analysis based on Benjamini-Hochberg corrected Fisher tests against some of the compound databases available in DAVID. Results are shown for (A) 979 genes mapping to 68 entries of Gene Ontology “Biological Process” (GO_BP), (B) 1,023 genes mapping to 104 entries of Gene Ontology “Molecular Function” (GO_MF), (C) 522 genes mapping to 36 entries of KEGG Pathway, (D) 1244 genes mapping to 120 entries of InterPro. (E, F, G, H) 1,652 genes were significantly downregulated by cytokines in at least 4 out of 5 islet samples, and significantly up-regulated in none. They were mapped to 1,620 unique entries in the DAVID database: (E) 1,151 genes mapping to 188 entries of Gene Ontology “Biological Process”, (B) 1,111 genes mapping to 57 entries of Gene Ontology “Molecular Function”, (C) 462 genes mapping to 25 entries of KEGG Pathway, (D) 1421 genes mapping to 94 entries of InterPro. The length of the grey bars indicates the significance of the association between the set of genes and the entry name, expressed as minus the logarithm of the probability that a set of genes taken at random from the human genome would be associated with the same entry. Only the 30 top entries are displayed. The red vertical line indicates a probability threshold of 0.05 (corresponding to a −log(BH p-value) of 1.3).
(TIF)

Figure S4 Protein–protein interaction analysis of cytokine up-regulated genes. 1,416 genes were significantly up-regulated by the cytokines IL-1β+IFN-γ in at least 4 out of 5 islet samples, and significantly downregulated in none. These genes were mapped to 1,403 unique entries in the BioProfiling database, and 55 of these entries were assembled into a unique network using as connecting nodes protein-protein interactions documented in the IntAct database. A representative figure is shown. A gene set enrichment analysis was performed and genes were color-coded to indicate association with the indicated Gene Ontology terms.
(TIF)

Figure S5 RT-PCR validation of the modulation of alternative splicing by cytokines in human islets. (A) Schematic representation of *DNAJA3* splice products amplified by RT-PCR, resulting in PCR products of 267 bp for variant 1 and 150 bp for variant 2. (B) Relative abundance of variants 1 and 2 was evaluated in three human islet preparations under control condition (Hi) or following exposure to the cytokines IL-1β+IFN-γ (Hi+Cyt).
(TIF)

Table S1 Sequence of the primers used in this study. STD: primers used for conventional PCR, qRT: primers used for real time qRT-PCR. The RefSeq ID of the sequence used to design the primers is provided.
(DOC)

Table S2 Mapping and quantification statistics for the RNA-seq data. Sequencing reads for 5 human islet samples cultured under control conditions were mapped to the human genome using GEM. Only a fraction of the total number of reads could be mapped. The number of mappings is greater than the number of mapped reads since some reads were mapped to more than one alternative location. The mappings were subsequently “paired” onto the RefSeq annotated transcripts using Flux Capacitor. Only a fraction of the reads could be paired. The number of paired mappings ( = number of transcript counts) is greater than the number of paired reads since sometimes it is not possible to choose between alternative transcripts. The last column gives the number of genetic loci for which at least one read is paired to one transcript.
(DOC)

Table S3 Expression of T1D candidate genes in human islets under control and pro-inflammatory conditions. For these T1D candidate genes, RNA-seq gene expression is provided in 5 human islet preparations (see Table 1) cultured under control condition or following exposure to the pro-inflammatory cytokines IL-1β+IFN-γ, mimicking inflammation. The sum of the RPKM for all the transcripts of the same gene is taken as measure of gene expression and the median of the 5 values is provided. Genes that were not detected or had an RPKM<1 for either condition are not mentioned.
(DOC)

Table S4 Expression of genes involved in radical scavenging in human islets and other tissues. For a number of genes known to be involved in radical scavenging a comparison is made between the 5 studied human islet preparations (see Table 1) cultured under control conditions and 5 selected tissues from the Illumina Human Body Map (adipose tissue, colon, kidney, liver and skeletal muscle). The sum of the RPKM for all the transcripts from the same gene is taken as measure of gene expression. The third column contains the median of the expression values for the 5 human islet samples. The log_2_ of the proportion between the level of gene expression for an islet preparation and the level of gene expression for a background tissue is taken as the measure of difference in gene expression. The last 5 columns contain the median of the significant differences in gene expression between the 5 islet preparations and a background tissue. If there is a significant difference (a description of the statistical analysis is provided in Materials and Methods) in one direction for at least 4 out of 5 islet samples and in the other direction for none the value is considered significant and shown in bold font, otherwise it is considered non-significant.
(DOC)

Table S5 Classification of selected IL-1β+IFN-γ-modulated genes in human islet cells into functional groups. For a selected number of genes a comparison is shown between the 5 studied islet samples (see Table 1) cultured under control conditions and in the presence of cytokines (IL-1β+IFN-γ). The log_2_ of the proportion between the sum of the RPKM for all the transcripts from the same gene under cytokine treatment and the same sum obtained under control conditions was taken as measure of change in gene expression. A difference in gene expression was considered significant if the corrected p value<0.05. The table contains the median of the significant changes in gene expression. With a few exceptions genes were only taken up in the list when they were significantly changed in expression in one direction for at least 4 islet samples and changed in the other direction for none. *JUNB* and *SH2B3* were significantly downregulated and *BMF* was significantly up-regulated in 1 islet preparation but they were added to this list for the sake of completeness.
(DOC)
