# Supplementary material for: The Human Pancreatic Islet Transcriptome: Expression of Candidate Genes for Type 1 Diabetes and the Impact of Pro-Inflammatory Cytokines
Source: PLoS Genet. 2012 Mar 8;8(3):e1002552. doi: 10.1371/journal.pgen.1002552 (PMC3297576; doi:10.1371/journal.pgen.1002552)
Supplement: Dataset S1 — RPKM data and lists of cytokine-modified and human islet-specific genes. Includes “table_RPKM.xlsx” (detected transcripts with their expression levels), “ctrl_cyt_expr.up.xlsx” (list of upregulated genes), “ctrl_cyt_expr.down.xlsx” (list of downregulated genes), “ctrl_cyt_AS_up.xlsx” (list of upregulated splicing isoforms), “ctrl_cyt_AS_down.xlsx” (list of downregulated splicing isoforms) and “Legends.docx” (explanation of the tables). (ZIP) [file pgen.1002552.s001.zip › Legends.docx]

**table_RPKM**

**RPKM values for islet preparations cultured under control conditions versus cytokine-treatment** : Langerhans islets were isolated from 5 different human organ donors and were cultured either under control conditions or in the presence of cytokines (IL‑1β + IFN‑γ). Reads were obtained by total RNA extraction followed by Illumina sequencing. RPKM values were determined by mapping the reads against the human genome using GEM and then “pairing” the reads onto the annotated transcripts from the RefSeq databank using Flux Capacitor.

**ctl_cyt_expr_up**

**ctl_cyt_expr_down**

**Lists with genes that are up- or downregulated in islets after cytokine treatment** : A comparison is made between 5 islet samples cultured under control conditions and in the presence of cytokines (IL‑1β + IFN‑γ). The log_2_ of the proportion between the sum of the RPKM for all the transcripts from the same gene under cytokine treatment and the same sum obtained under control conditions is taken as measure of change in gene expression. The p‑value is obtained by performing a Fisher exact test (number of reads mapped to gene and number of reads mapped to all other genes versus number of reads obtained under cytokine treatment and number of reads obtained under control conditions) and is corrected by the Benjamini-Hochberg method (taking for each gene the 5 samples as independent tests). A difference in gene expression is considered significant if the corrected p-value > 0.05. A gene is taken up in a list only if its expression is changed significantly in one direction in at least 4 out of 5 islet samples and in the other direction in none. An “existence score” is computed as the arithmetic mean of the natural logarithms of the corrected p-values.

**ctl_cyt_AS_up**

**ctl_cyt_AS_down**

**Lists with splicing isoforms that are up- or downregulated in islets after cytokine treatment** : A comparison is made between 5 islet samples cultured under control conditions and in the presence of cytokines (IL‑1β + IFN‑γ). The proportion between the RPKM for a transcript and the sum of the RPKM for all the transcripts from the same gene is taken and the delta value is computed as the difference between this proportion obtained under cytokine treatment and the same proportion obtained under control conditions. The p-value is obtained by performing a Fisher exact test (number of reads mapped to transcript and number of reads mapped to all other transcripts of the same gene versus number of reads obtained under cytokine treatment and number of reads obtained under control conditions) and is corrected by the Benjamini-Hochberg method (taking for each transcript the 5 samples as independent tests). A change in alternative splicing is considered significant if the corrected p-value > 0.05. A transcript is taken up in a list only if its splicing is changed significantly in one direction in at least 4 out of 5 islet samples and in the other direction in none. An “existence score” is computed as the arithmetic mean of the natural logarithms of the corrected p-values.
