# Supplementary figures and images for: The Human Pancreatic Islet Transcriptome: Expression of Candidate Genes for Type 1 Diabetes and the Impact of Pro-Inflammatory Cytokines
Source: PLoS Genet. 2012 Mar 8;8(3):e1002552. doi: 10.1371/journal.pgen.1002552 (PMC3297576; doi:10.1371/journal.pgen.1002552)

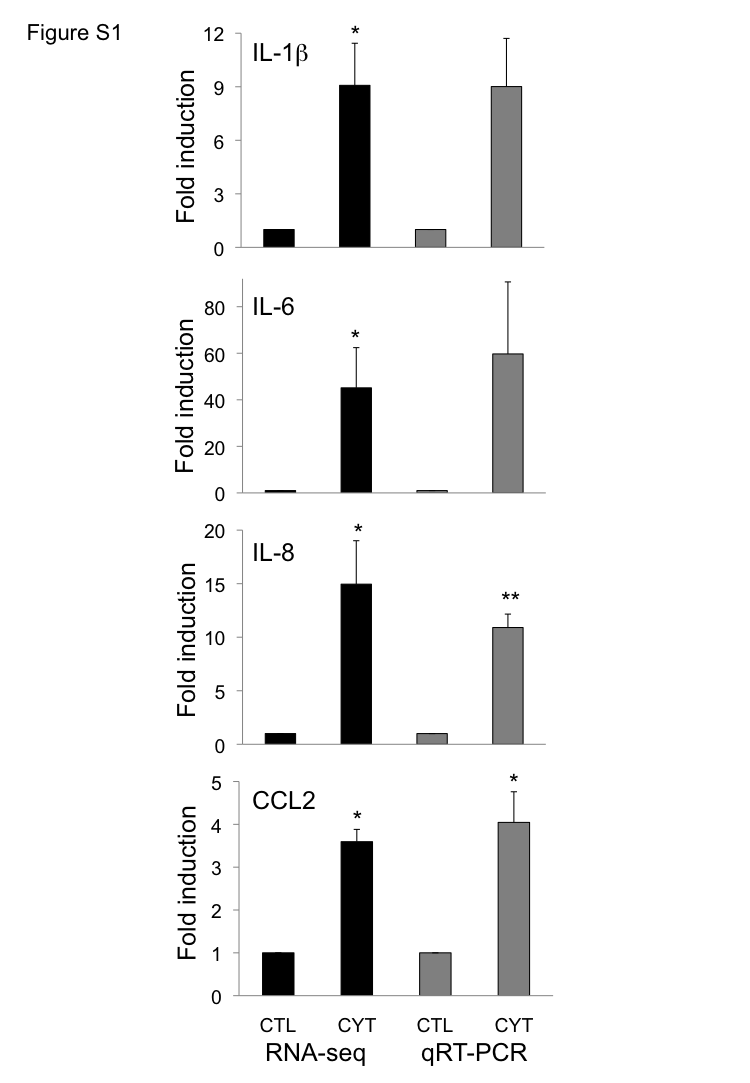

Supplement: Figure S1 — Validation of RNA-seq gene expression data by qRT-PCR in cytokine-treated human islets. Human islets from 5 organ donors were cultured for 48 h in the presence (CYT) or absence (CTL) of the cytokines IL-1β+IFN-γ. RNA-seq gene expression results (black bars) were compared to gene expression assessed by qRT-PCR (gray bars) in the 5 human islet preparations used for RNA-seq. Data were normalized to the geometric mean of β-actin and GAPDH expression and expressed as fold induction of control. *p<0.05, **p<0.01 for CYT versus CTL. (TIF) [file pgen.1002552.s002.tif]

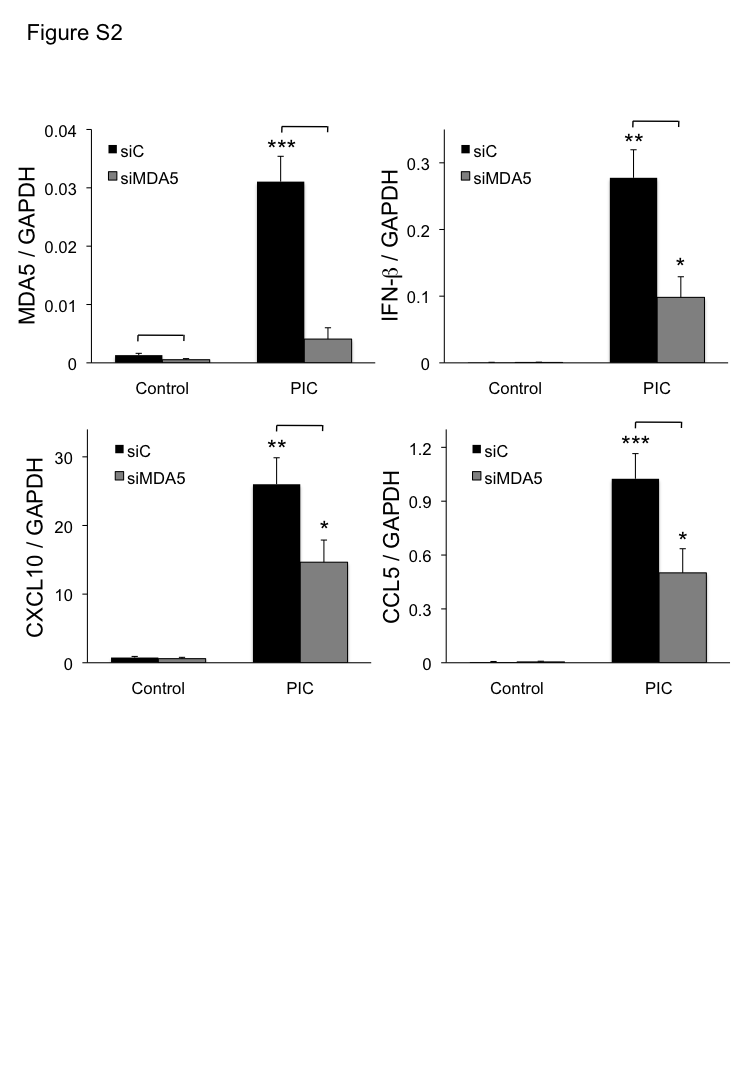

Supplement: Figure S2 — MDA5 regulates cytokine and chemokine production in primary rat beta cells exposed to intracellular dsRNA. FACS-purified rat beta cells were transfected with control siRNA (siC, black bars) or siRNA targeting MDA5 (siMDA5, grey bars). After 48 h, cells were left untreated or transfected with PIC for 48 h. MDA5, IFN-β, CCL5 and CXCL10 mRNA expression was assayed by qRT-PCR and corrected for the housekeeping gene GAPDH. Results are mean ± SEM of six independent experiments. *p<0.05, **p<0.01, ***p<0.001 versus control; p<0.05 for the comparison siC versus siMDA5 as indicated. (TIF) [file pgen.1002552.s003.tif]

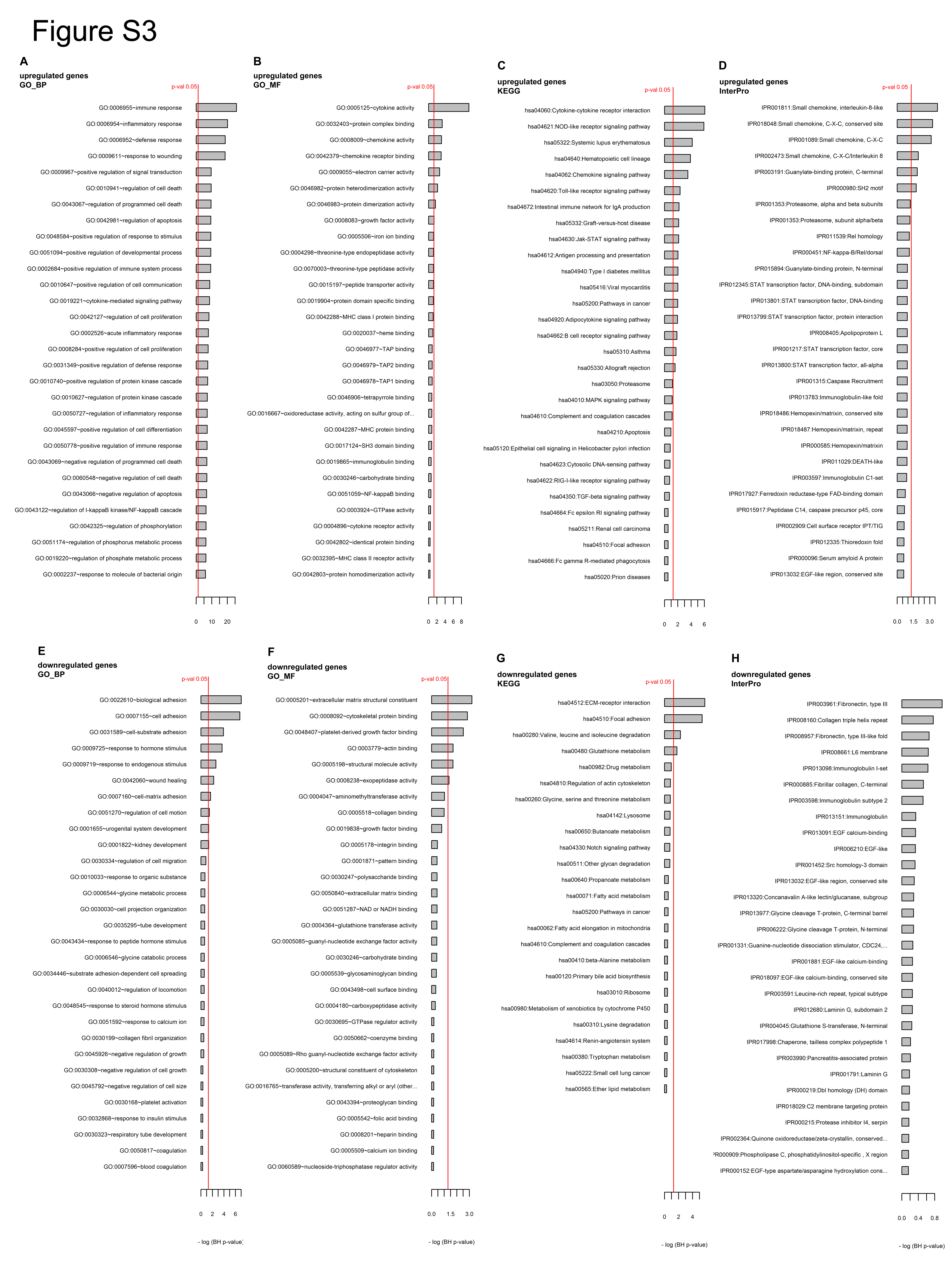

Supplement: Figure S3 — DAVID analysis of cytokine-modified genes. (A, B, C, D) 1,416 genes were significantly up-regulated by the cytokines IL-1β+IFN-γ in at least 4 out of 5 islet samples, and significantly downregulated in none. These genes mapped to 1,395 unique entries in the DAVID database, which were submitted to gene set enrichment analysis based on Benjamini-Hochberg corrected Fisher tests against some of the compound databases available in DAVID. Results are shown for (A) 979 genes mapping to 68 entries of Gene Ontology “Biological Process” (GO_BP), (B) 1,023 genes mapping to 104 entries of Gene Ontology “Molecular Function” (GO_MF), (C) 522 genes mapping to 36 entries of KEGG Pathway, (D) 1244 genes mapping to 120 entries of InterPro. (E, F, G, H) 1,652 genes were significantly downregulated by cytokines in at least 4 out of 5 islet samples, and significantly up-regulated in none. They were mapped to 1,620 unique entries in the DAVID database: (E) 1,151 genes mapping to 188 entries of Gene Ontology “Biological Process”, (B) 1,111 genes mapping to 57 entries of Gene Ontology “Molecular Function”, (C) 462 genes mapping to 25 entries of KEGG Pathway, (D) 1421 genes mapping to 94 entries of InterPro. The length of the grey bars indicates the significance of the association between the set of genes and the entry name, expressed as minus the logarithm of the probability that a set of genes taken at random from the human genome would be associated with the same entry. Only the 30 top entries are displayed. The red vertical line indicates a probability threshold of 0.05 (corresponding to a −log(BH p-value) of 1.3). (TIF) [file pgen.1002552.s004.tif]

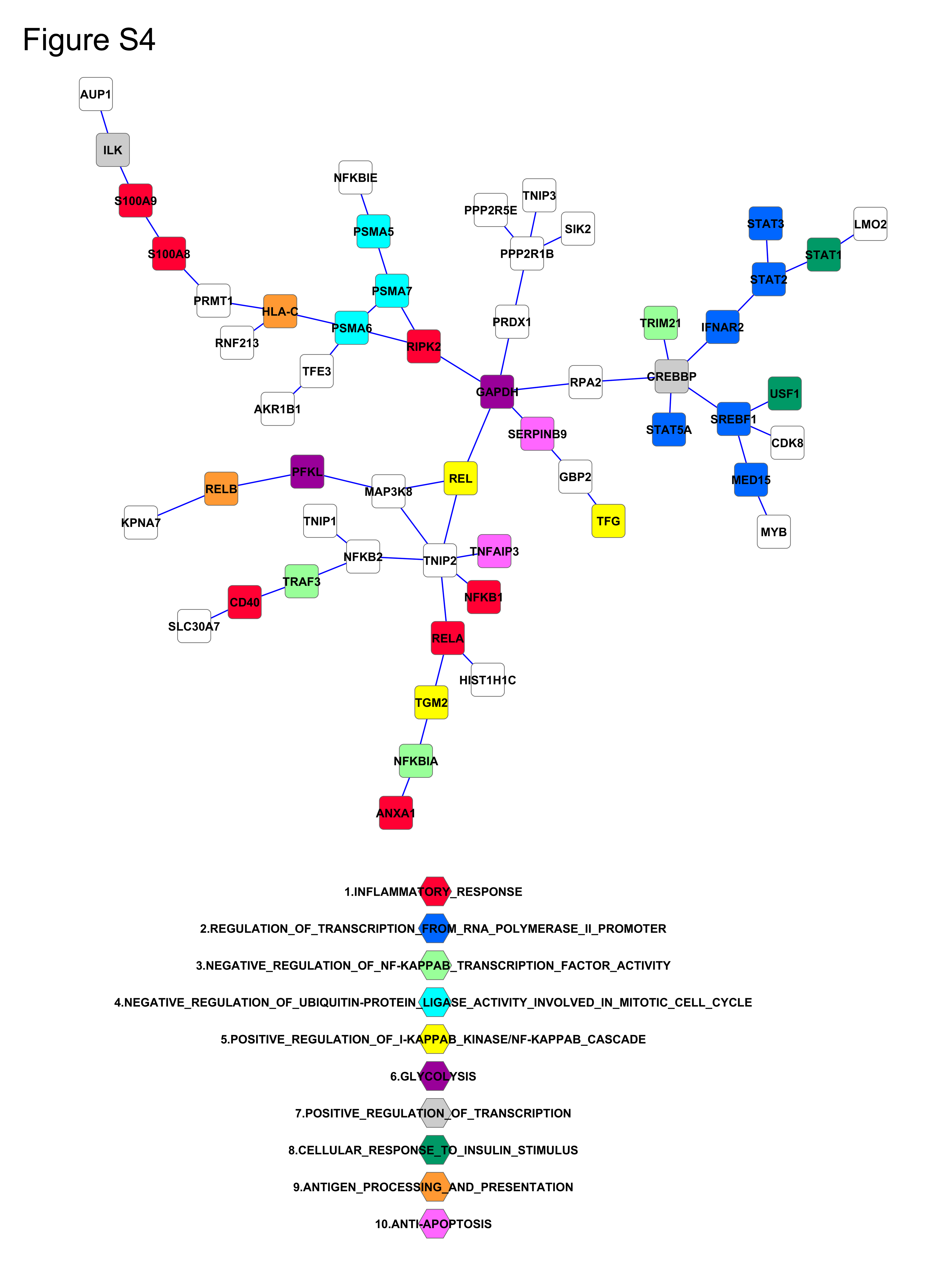

Supplement: Figure S4 — Protein–protein interaction analysis of cytokine up-regulated genes. 1,416 genes were significantly up-regulated by the cytokines IL-1β+IFN-γ in at least 4 out of 5 islet samples, and significantly downregulated in none. These genes were mapped to 1,403 unique entries in the BioProfiling database, and 55 of these entries were assembled into a unique network using as connecting nodes protein-protein interactions documented in the IntAct database. A representative figure is shown. A gene set enrichment analysis was performed and genes were color-coded to indicate association with the indicated Gene Ontology terms. (TIF) [file pgen.1002552.s005.tif]

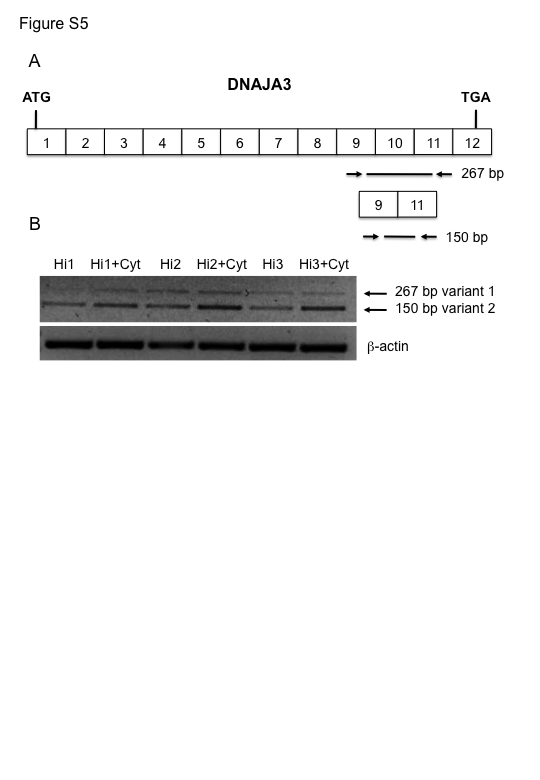

Supplement: Figure S5 — RT-PCR validation of the modulation of alternative splicing by cytokines in human islets. (A) Schematic representation of DNAJA3 splice products amplified by RT-PCR, resulting in PCR products of 267 bp for variant 1 and 150 bp for variant 2. (B) Relative abundance of variants 1 and 2 was evaluated in three human islet preparations under control condition (Hi) or following exposure to the cytokines IL-1β+IFN-γ (Hi+Cyt). (TIF) [file pgen.1002552.s006.tif]
