# Supplementary material for: The Human Pancreatic Islet Transcriptome: Expression of Candidate Genes for Type 1 Diabetes and the Impact of Pro-Inflammatory Cytokines
Source: PLoS Genet. 2012 Mar 8;8(3):e1002552. doi: 10.1371/journal.pgen.1002552 (PMC3297576; doi:10.1371/journal.pgen.1002552)
Supplement: Table S1 — Sequence of the primers used in this study. STD: primers used for conventional PCR, qRT: primers used for real time qRT-PCR. The RefSeq ID of the sequence used to design the primers is provided. (DOC) [file pgen.1002552.s007.doc]

**Table S1: Sequence of the primers used in this study.**

| Gene | Species | RefSeq ID | STD or qRT | Forward primer | Reverse primer | Product length (bp) |
| --- | --- | --- | --- | --- | --- | --- |
| ACTB | Homo sapiens | NM_001101 | STD | AAATCTGGCACCACACCTTC | CCGATCCACACGGAGTACTT | 805 |
|  |  |  | qRT | CTGTACGCCAACACAGTGCT | GCTCAGGAGGAGCAATGATC | 127 |
| BACH2 | Rattus norvegicus | NM_001135754 | STD | CACGGCGGACTGATGGCTGA | TGGCTCCAGGCAGCAGGGTA | 870 |
|  |  |  | qRT | GATCACAGACCTTCCCAGGA | TCTCTTTCTCGCACACCAGTT | 196 |
| BCL2A1 | Homo sapiens | NM_004049 | STD | GCTCAGGACTATCTGCAGTGCGT | AAAGCCATTTTCCCAGCCTCCG | 396 |
|  |  |  | qRT | TGTCCGTAGACACTGCCAGAACA | TCCGGGGCAATTTGCTGTCG | 148 |
| BCL2A1 | Rattus norvegicus | NM_133416.1 | STD | CAGCAGAATGGAGGCTGG | CCAGGGTTCTCTCTGGTC | 142 |
|  |  |  | qRT | GGCTGGGAAGATGGCTTC | CCAGGGTTCTCTCTGGTC | 130 |
| BMF | Homo sapiens | NM_001003940 | STD | GGAGCCCTGGCATCACGACTCG | GCCGGTGGAACTGGTCTGCAAT | 497 |
|  |  |  | qRT | GACCCAACCCGGGAGCTTGC | GAAGGCCAGGGCCACAGCAG | 113 |
| CCL2 | Homo sapiens | NM_002982 | STD | TTCTGTGCCTGCTGCTCATA | GTCTTCGGAGTTTGGGTTTG | 277 |
|  |  |  | qRT | AGCAAGTGTCCCAAAGAAGC | CATGGAATCCTGAACCCACT | 93 |
| CCL5 | Rattus norvegicus | NM_031116.3 | STD | GCTGCTTTGCCTACCTCTC | ATCTATGCCCTCCCAGGAAT | 302 |
|  |  |  | qRT | CCAGAGAAGAAGTGGGTTCA | AGCAAGCAA TGACAGGAAAG | 104 |
| CXCL10 | Rattus norvegicus | NM_139089.1 | STD | GAAGCACCA TGAACCCAAGT | GCAAGTCTATCCTGTCCGCAT | 380 |
|  |  |  | qRT | GGGTAAAGGGAGGTGGAGAG | GGGTAAAGGGAGGTGGAGAGA | 216 |
| DNAJA3 | Homo sapiens | NM_005147 | STD | GGGACGGTGAACGGCGTCA | AGAGGCTCTGCGGGTGGGAG | 267 |
|  |  | NM_001135110 | STD | GGGACGGTGAACGGCGTCA | AGAGGCTCTGCGGGTGGGAG | 150 |
| GABRG2 | Rattus norvegicus | NM_183327 | qRT | AGCCCGGAAGTCTCTGCCCA | CCCGTGTCTCCAGGCTCCTGT | 334 |
| GAPDH | Rattus norvegicus | NM_017008 | STD | ATGACTCTACCCACGGCAAG | TGTGAGGGAGATGCTCAGTG | 975 |
|  |  |  | qRT | AGTTCAACGGCACAGTCAAG | TACTCAGCACCAGCATCACC | 118 |
| IFNB | Rattus norvegicus | NM_019127.1 | STD | CTGCCCTCTCCATCGACTAC | TCCGAGCAGAAGTCTT | 453 |
|  |  |  | qRT | GCCTTTGCCATTCAAG | AGACAGAGCTTCTGGA | 131 |
| IL1B | Homo sapiens | NM_000576 | STD | GCTGAGGAAGATGCTGGTTC | TTCTGCTTGAGAGGTGCTGA | 514 |
|  |  |  | qRT | TCCAGGGACAGGATATGGAG | TCTTTCAACACGCAGGACAG | 133 |
| IL6 | Homo sapiens | NM_000600 | STD | AGTACCCCCAGGAGAAGATT | TACTCATCTGCACAGCTCTG | 354 |
|  |  |  | qRT | AAAAGATGGCTGAAAAAGATGG | CTACTCTCAAATCTGTTCTGG | 129 |
| IL8 | Homo sapiens | NM_000584 | STD | AGGAAGAAACCACCGGAAG | TCTTCAAAAACTTCTCCACAAC | 325 |
|  |  |  | qRT | TGTAAACATGACTTCCAAGCT | TTGGAGTATGTCTTTATGCAC | 131 |
| MDA5 | Rattus norvegicus | NM_001109199.1 | STD | TGACGAGTGTCTCCACTTGC | TCCATTTGGTAAGGCCTGAG | 612 |
|  |  |  | qRT | TGTCTTGGQCQCTTGCTTCG | TGCTGAGAAGGA TTGTGCAG | 121 |
| NOVA1 | Homo sapiens | NM_002515 | STD | TGCAGCTGCTCCTCAGCCCT | ACTGGCCGTCTTCGCCCGTA | 363 |
|  |  |  | qRT | CCGGTAGCAGCGGCAGGAAC | AGCGGCCTTTTCCGCGAGTC | 116 |
| NOVA1 | Rattus norvegicus | XM_234098 | STD | TCTGACCCCATGACCACCTCCA | CTGCTGGGAAGGCCGCAACA | 421 |
|  |  |  | qRT | CCACAGAGTGGCAGCTGTCTCAA | CTGCTGGGAAGGCCGCAACA | 175 |
| SH2B3 | Rattus norvegicus | NM_031621 | STD | TCTGCGGGTCACCACCACCA | CGCTGGAAGTGGCGGAGCAT | 339 |
|  |  |  | qRT | ACGGTGTGTTCCTGGTGCGG | AGGCGGAGGTGCTTGGCTCT | 91 |

STD: primers used for conventional PCR, qRT: primers used for real time qRT-PCR. The RefSeq ID of the sequence used to design the primers is provided.
