# Supplementary material for: The Human Pancreatic Islet Transcriptome: Expression of Candidate Genes for Type 1 Diabetes and the Impact of Pro-Inflammatory Cytokines
Source: PLoS Genet. 2012 Mar 8;8(3):e1002552. doi: 10.1371/journal.pgen.1002552 (PMC3297576; doi:10.1371/journal.pgen.1002552)
Supplement: Table S2 — Mapping and quantification statistics for the RNA-seq data. Sequencing reads for 5 human islet samples cultured under control conditions were mapped to the human genome using GEM. Only a fraction of the total number of reads could be mapped. The number of mappings is greater than the number of mapped reads since some reads were mapped to more than one alternative location. The mappings were subsequently “paired” onto the RefSeq annotated transcripts using Flux Capacitor. Only a fraction of the reads could be paired. The number of paired mappings ( = number of transcript counts) is greater than the number of paired reads since sometimes it is not possible to choose between alternative transcripts. The last column gives the number of genetic loci for which at least one read is paired to one transcript. (DOC) [file pgen.1002552.s008.doc]

Table S2: Mapping and quantification statistics for the RNA-seq data.

| Sample | Reads | Mapped reads | Mappings | Paired reads | Paired mappings | Loci |
| --- | --- | --- | --- | --- | --- | --- |
| ID1 | 13,570,474 | 11,040,219 | 17,121,512 | 6,079,142 | 8,626,757 | 16,399 |
| ID2 | 59,330,966 | 51,481,529 | 75,595,703 | 28,943,696 | 41,010,797 | 18,094 |
| ID3 | 57,405,514 | 51,374,139 | 76,278,742 | 28,914,532 | 47,830,760 | 18,084 |
| ID4 | 23,076,808 | 18,995,344 | 26,817,392 | 10,234,664 | 11,596,502 | 17,382 |
| ID5 | 51,435,444 | 39,577,652 | 57,079,480 | 19,123,192 | 22,788,527 | 17,787 |

Sequencing reads for 5 human islet samples cultured under control conditions were mapped to the human genome using GEM. Only a fraction of the total number of reads could be mapped. The number of mappings is greater than the number of mapped reads since some reads were mapped to more than one alternative location. The mappings were subsequently “paired” onto the RefSeq annotated transcripts using Flux Capacitor. Only a fraction of the reads could be paired. The number of paired mappings (= number of transcript counts) is greater than the number of paired reads since sometimes it is not possible to choose between alternative transcripts. The last column gives the number of genetic loci for which at least one read is paired to one transcript.
