# Supplementary material for: The Human Pancreatic Islet Transcriptome: Expression of Candidate Genes for Type 1 Diabetes and the Impact of Pro-Inflammatory Cytokines
Source: PLoS Genet. 2012 Mar 8;8(3):e1002552. doi: 10.1371/journal.pgen.1002552 (PMC3297576; doi:10.1371/journal.pgen.1002552)
Supplement: Table S3 — Expression of T1D candidate genes in human islets under control and pro-inflammatory conditions. For these T1D candidate genes, RNA-seq gene expression is provided in 5 human islet preparations (see Table 1) cultured under control condition or following exposure to the pro-inflammatory cytokines IL-1β+IFN-γ, mimicking inflammation. The sum of the RPKM for all the transcripts of the same gene is taken as measure of gene expression and the median of the 5 values is provided. Genes that were not detected or had an RPKM<1 for either condition are not mentioned. (DOC) [file pgen.1002552.s009.doc]

**Table S3: Expression of T1D candidate genes in human islets under control and pro-inflammatory conditions.**

| Gene name | Gene description | Median sum RPKM control | Median sum RPKM cytokine |
| --- | --- | --- | --- |
| C1QTNF6 | C1q and tumor necrosis factor related protein 6 | 7.1 | 3.2 |
| CENPW | centromere protein W | 1.2 | 1.0 |
| CLEC16A | C-type lectin domain family 16, member A | 7.5 | 7.5 |
| COBL | cordon-bleu homolog (mouse) | 20 | 14 |
| CTRB1 | chymotrypsinogen B1 | 705 | 462 |
| CTRB2 | chymotrypsinogen B2 | 749 | 544 |
| CTSH | cathepsin H | 57 | 16 |
| CYP27B1 | cytochrome P450, family 27, subfamily B, polypeptide 1 | 0.8 | 2.3 |
| DLK1 | delta-like 1 homolog (Drosophila) | 151 | 386 |
| ERBB3 | v-erb-b2 erythroblastic leukemia viral oncogene homolog 3 (avian) | 29 | 22 |
| FUT2 | fucosyltransferase 2 (secretor status included) | 3.4 | 4.3 |
| GLIS3 | GLIS family zinc finger 3 | 13 | 13 |
| GPR183 | G protein-coupled receptor 183 | 1.3 | 0.6 |
| HLA-DQB1 | major histocompatibility complex, class II, DQ beta 1 | 5.48 | 14 |
| HLA-DRB1 | major histocompatibility complex, class II, DR beta 1 | 18 | 81 |
| IFIH1 | interferon induced with helicase C domain 1 | 14 | 20 |
| INS | insulin | 21626 | 24797 |
| ORMDL3 | ORM1-like 3 (S. cerevisiae) | 32 | 32 |
| PTPN2 | protein tyrosine phosphatase, non-receptor type 2 | 5.8 | 5.6 |
| RASGRP1 | RAS guanyl releasing protein 1 (calcium and DAG-regulated) | 3.7 | 7.2 |
| RNLS | renalase, FAD-dependent amine oxidase | 1.8 | 1.4 |
| SH2B3 | SH2B adaptor protein 3 | 9.3 | 12 |
| SKAP2 | src kinase associated phosphoprotein 2 | 6.2 | 3.9 |
| SMARCE1 | SWI/SNF related, matrix associated, actin dependent regulator of chromatin, subfamily e, member 1 | 26 | 54 |
| STAT4 | signal transducer and activator of transcription 4 | 3.4 | 6.5 |
| TNFAIP3 | tumor necrosis factor, alpha-induced protein 3 | 27 | 79 |
| TYK2 | tyrosine kinase 2 | 23 | 20 |

For these T1D candidate genes, RNA-seq gene expression is provided in 5 human islet preparations (see Table 1) cultured under control condition or following exposure to the pro-inflammatory cytokines IL-1β + IFN-γ, mimicking inflammation. The sum of the RPKM for all the transcripts of the same gene is taken as measure of gene expression and the median of the 5 values is provided. Genes that were not detected or had an RPKM <1 for either condition are not mentioned.
