# Supplementary material for: The Human Pancreatic Islet Transcriptome: Expression of Candidate Genes for Type 1 Diabetes and the Impact of Pro-Inflammatory Cytokines
Source: PLoS Genet. 2012 Mar 8;8(3):e1002552. doi: 10.1371/journal.pgen.1002552 (PMC3297576; doi:10.1371/journal.pgen.1002552)
Supplement: Table S4 — Expression of genes involved in radical scavenging in human islets and other tissues. For a number of genes known to be involved in radical scavenging a comparison is made between the 5 studied human islet preparations (see Table 1) cultured under control conditions and 5 selected tissues from the Illumina Human Body Map (adipose tissue, colon, kidney, liver and skeletal muscle). The sum of the RPKM for all the transcripts from the same gene is taken as measure of gene expression. The third column contains the median of the expression values for the 5 human islet samples. The log2 of the proportion between the level of gene expression for an islet preparation and the level of gene expression for a background tissue is taken as the measure of difference in gene expression. The last 5 columns contain the median of the significant differences in gene expression between the 5 islet preparations and a background tissue. If there is a significant difference (a description of the statistical analysis is provided in Materials and Methods) in one direction for at least 4 out of 5 islet samples and in the other direction for none the value is considered significant and shown in bold font, otherwise it is considered non-significant. (DOC) [file pgen.1002552.s010.doc]

**Table S4: Expression of genes involved in radical scavenging in human islets and other tissues.**

| Gene name | Gene description | Median sum RPKM islets | Median log_2_FC adipose | Median log_2_FC colon | Median log_2_FC kidney | Median log_2_FC liver | Median log_2_FC muscle |
| --- | --- | --- | --- | --- | --- | --- | --- |
| CAT | catalase | 26 | **-1.92** | **-0.99** | **-1.53** | **-0.39** | 0.28 |
| SOD1 | superoxide dismutase 1, soluble | 73 | 0.22 | **-0.35** | **-0.34** | 0.17 | **1.71** |
| SOD2 | superoxide dismutase 2, mitochondrial | 388 | **-1.54** | -0.60 | **-1.72** | **1.29** | **-4.97** |
| SOD3 | superoxide dismutase 3, extracellular | 24 | **-2.59** | **-1.88** | **1.65** | **6.40** | **1.91** |
| GPX1 | glutathione peroxidase 1 isoform 1 | 63 | **-0.80** | -0.42 | **-1.16** | **-1.02** | **2.93** |
| GPX2 | glutathione peroxidase 2 (gastrointestinal) | 104 | **9.06** | **3.28** | **2.15** | 0.78 | **Inf** |
| GPX3 | glutathione peroxidase 3 (plasma) | 141 | **-4.27** | **-3.19** | **-4.60** | -0.74 | **3.44** |
| GPX4 | glutathione peroxidase 4 (phospholipid hydroperoxidase) | 98 | **-1.83** | **-0.21** | -0.07 | **-0.75** | **1.48** |
| GPX7 | glutathione peroxidase 7 | 5 | **1.24** | **1.75** | **0.98** | **3.76** | **3.56** |
| GPX8 | glutathione peroxidase 8 (putative) | 5 | 0.36 | 0.26 | -0.59 | **4.52** | **3.75** |

For a number of genes known to be involved in radical scavenging a comparison is made between the 5 studied human islet preparations (see Table 1) cultured under control conditions and 5 selected tissues from the Illumina Human Body Map (adipose tissue, colon, kidney, liver and skeletal muscle). The sum of the RPKM for all the transcripts from the same gene is taken as measure of gene expression. The third column contains the median of the expression values for the 5 human islet samples. The log_2_ of the proportion between the level of gene expression for an islet preparation and the level of gene expression for a background tissue is taken as the measure of difference in gene expression. The last 5 columns contain the median of the significant differences in gene expression between the 5 islet preparations and a background tissue. If there is a significant difference (a description of the statistical analysis is provided in Materials and Methods) in one direction for at least 4 out of 5 islet samples and in the other direction for none the value is considered significant and shown in bold font, otherwise it is considered non-significant.
