# Supplementary material for: The Human Pancreatic Islet Transcriptome: Expression of Candidate Genes for Type 1 Diabetes and the Impact of Pro-Inflammatory Cytokines
Source: PLoS Genet. 2012 Mar 8;8(3):e1002552. doi: 10.1371/journal.pgen.1002552 (PMC3297576; doi:10.1371/journal.pgen.1002552)
Supplement: Table S5 — Classification of selected IL-1β+IFN-γ-modulated genes in human islet cells into functional groups. For a selected number of genes a comparison is shown between the 5 studied islet samples (see Table 1) cultured under control conditions and in the presence of cytokines (IL-1β+IFN-γ). The log2 of the proportion between the sum of the RPKM for all the transcripts from the same gene under cytokine treatment and the same sum obtained under control conditions was taken as measure of change in gene expression. A difference in gene expression was considered significant if the corrected p value<0.05. The table contains the median of the significant changes in gene expression. With a few exceptions genes were only taken up in the list when they were significantly changed in expression in one direction for at least 4 islet samples and changed in the other direction for none. JUNB and SH2B3 were significantly downregulated and BMF was significantly up-regulated in 1 islet preparation but they were added to this list for the sake of completeness. (DOC) [file pgen.1002552.s011.doc]

**Table S5: Classification of selected IL-1β + IFN-γ-modulated genes in human islet cells into functional groups.**

| Gene name | Gene description | Log_2_ fold change |
| --- | --- | --- |
| **NO formation** | | |
| NOS2 | nitric oxide synthase 2, inducible | 7.09 |
| **Glucose metabolism/Kreb’s cycle** | | |
| G6PC2 | glucose-6-phosphatase, catalytic, 2 | -1.89 |
| HK2 | hexokinase 2 | 1.99 |
| IDH2 | isocitrate dehydrogenase 2 (NADP+), mitochondrial | -0.95 |
| LDHA | lactate dehydrogenase A | 0.90 |
| PFKFB3 | 6-phosphofructo-2-kinase/fructose-2,6-biphosphatase 3 | 1.25 |
| PGK1 | phosphoglycerate kinase 1 | 0.76 |
| **Lipid metabolism** | | |
| ACSL4 | acyl-CoA synthetase long chain family member 4 | 1.29 |
| ACSL5 | acyl-CoA synthetase long chain family member 5 | 1.85 |
| ACSS1 | acyl-CoA synthetase short chain family member 1 | -1.25 |
| **IFN-γ signaling** | | |
| IRF1 | interferon regulatory factor 1 | 2.87 |
| IRF8 | interferon regulatory factor 8 | 2.45 |
| JAK2 | Janus kinase 2 | 1.35 |
| SOCS1 | suppressor of cytokine signaling 1 | 2.88 |
| SOCS3 | suppressor of cytokine signaling 3 | 2.03 |
| STAT1 | signal transducer and activator of transcription 1 | 2.71 |
| **NF-κB regulation** | | |
| IKBKE | inhibitor of kappa light polypeptide gene enhancer in B-cells, kinase, ε | 1.40 |
| IRAK2 | interleukin-1 receptor-associated kinase 2 | 1.04 |
| NFKB2 | nuclear factor of kappa light polypeptide gene enhancer in B-cells 2, p49/p100 | 1.33 |
| NFKBIA | nuclear factor of kappa light polypeptide gene enhancer in B-cells inhibitor, α | 2.34 |
| NFKBIZ | nuclear factor of kappa light polypeptide gene enhancer in B-cells inhibitor, ζ | 0.86 |
| TNIP1 | TNFAIP3 interacting protein 1 | 0.72 |
| TNIP2 | TNFAIP3 interacting protein 2 | 0.73 |
| TNIP3 | TNFAIP3 interacting protein 3 | 6.51 |
| **Other transcription factors** | | |
| BATF2 | basic leucine zipper transcription factor, ATF-like 2 | 3.44 |
| CEBPB | CCAAT/enhancer binding protein (C/EBP), β | 2.10 |
| DDX17 | probable ATP-dependent RNA helicase DDX17 | -0.39 |
| HIF1A | hypoxia inducible factor 1, α subunit | 0.54 |
| ID1 | inhibitor of DNA binding 1 | 2.85 |
| ID2B | inhibitor of DNA binding 2B | 1.56 |
| ID3 | inhibitor of DNA binding 3 | 1.24 |
| JUNB | jun B proto-oncogene | 1.71 |
| LIF | leukemia inhibitory factor | 2.27 |
| SBNO2 | strawberry notch homolog 2 (Drosophila) | 1.37 |
| **Hormones/growth factors/receptors** | | |
| CHGA | chromogranin A | 0.81 |
| GCGR | glucagon receptor | 1.29 |
| IAPP | islet amyloid polypeptide | -1.00 |
| **Protein synthesis/translation regulation/endoplasmic reticulum** | | |
| EIF3L | eukaryotic translation initiation factor 3, subunit L | -1.46 |
| ERO1L | ERO1-like (S. cerevisiae) | 0.98 |
| ERP27 | endoplasmic reticulum protein 27 | -3.90 |
| WARS | tryptophanyl-tRNA synthetase | 3.37 |
| **Kinases/phosphatases** | | |
| AKAP7 | A kinase anchor protein 7 | -1.95 |
| DUSP1 | dual specificity phosphatase 1 | 1.15 |
| PPP1R1B | protein phosphatase 1, regulatory subunit 1B | -1.73 |
| **Chemokines/cytokines/adhesion molecules and related proteins** | | |
| CCL2 | chemokine (C-C motif) ligand 2, Mcp-1 | 2.21 |
| CCL3 | chemokine (C-C motif) ligand 3, Mip-1α | 3.33 |
| CCL3L3 | chemokine (C-C motif) ligand 3-like 3 | 3.60 |
| CCL5 | chemokine (C-C motif) ligand 5, Rantes | 2.84 |
| CCL8 | chemokine (C-C motif) ligand 8, Mcp-2 | Inf |
| CCL22 | chemokine (C-C motif) ligand 22 | 1.70 |
| CD40 | TNF receptor superfamily receptor member 5 | 1.66 |
| CD44 | CD44 molecule (Indian blood group) | -0.63 |
| CSF1 | colony stimulating factor 1 (macrophage) | 2.35 |
| CSF2 | colony stimulating factor 2 (granulocyte-macrophage) | 4.05 |
| CSF3 | colony stimulating factor 3 (granulocyte) | 7.87 |
| CXCL1 | chemokine (C-X-C motif) ligand 1, Gro-α | 4.18 |
| CXCL2 | chemokine (C-X-C motif) ligand 2, Gro-β | 3.00 |
| CXCL3 | chemokine (C-X-C motif) ligand 3, Gro-γ | 3.83 |
| CXCL5 | chemokine (C-X-C motif) ligand 5, ENA-78 | 4.96 |
| CXCL9 | chemokine (C-X-C motif) ligand 9, Mig | 5.52 |
| CXCL10 | chemokine (C-X-C motif) ligand 10, IP-10 | 7.07 |
| CXCL11 | chemokine (C-X-C motif) ligand 11, I-Tac | 4.98 |
| CX3CL1 | chemokine (C-X3-C motif) ligand 1, Fractalkine | 2.55 |
| ICAM1 | intercellular adhesion molecule 1 | 2.78 |
| ICAM4 | intercellular adhesion molecule 4, Landsteiner-Wiener blood group | 4.94 |
| IL1B | interleukin 1, β | 3.38 |
| IL1A | interleukin 1, α | 3.81 |
| IL6 | interleukin 6, interferon β2 | 5.45 |
| IL7 | interleukin 7 | 2.62 |
| IL8 | interleukin 8 | 3.70 |
| IL11 | interleukin 11 | 2.39 |
| IL15RA | interleukin 15 receptor α | 1.25 |
| IL17C | interleukin 17C | 3.31 |
| IL18BP | interleukin 18 binding protein | 3.55 |
| IL23A | interleukin 23, α subunit p19 | 3.99 |
| IL24 | interleukin 24 | 2.83 |
| IL32 | interleukin 32 | 0.96 |
| IL33 | interleukin 33 | 2.20 |
| LTB | lymphotoxin β | 3.13 |
| TNF | tumor necrosis factor | 2.69 |
| TNFAIP2 | tumor necrosis factor, α -induced protein 2 | 1.69 |
| TNFAIP3 | tumor necrosis factor, α -induced protein 3 | 1.33 |
| TNFAIP6 | tumor necrosis factor, α -induced protein 6 | 3.08 |
| TNFSF15 | tumor necrosis factor (ligand) superfamily, member 15 | 2.88 |
| **Other innate immune response/pro-inflammatory components** | | |
| CD55 | complement decay-accelerating factor | 0.45 |
| IDO1 | indoleamine 2,3-dioxygenase 1 | 8.05 |
| OAS1 | 2',5'-oligoadenylate synthetase 1 | 1.46 |
| OAS3 | 2',5'-oligoadenylate synthetase 3 | 1.84 |
| PTAFR | platelet-activating factor receptor | 3.12 |
| PTGES | prostaglandin E synthase | 1.71 |
| S100A7A | S100 calcium binding protein A7A | Inf |
| SH2B3 | SH2B adaptor protein 3 | 0.60 |
| TRAFD1 | TRAF-type zinc finger domain containing 1 | 0.95 |
| VNN2 | vanin 2 | 1.83 |
| **Proteasome/antigen presentation (HLA-related)** | | |
| PSMA5 | proteasome subunit, α type, 5 | 0.47 |
| PSMA6 | proteasome subunit, α type, 6 | 0.79 |
| PSMA7 | proteasome subunit, α type, 7 | 0.45 |
| PSMB8 | proteasome subunit, β type, 8 | 1.60 |
| PSMB9 | proteasome subunit, β type, 9 | 3.12 |
| PSMB10 | proteasome subunit, β type, 10 | 2.04 |
| PSME2 | proteasome activator subunit 2 | 1.32 |
| HLA-C | major histocompatibility complex, class I, C | 1.28 |
| HLA-DMA | major histocompatibility complex, class II, DMα | 1.24 |
| HLA-DMB | major histocompatibility complex, class II, DMβ | 1.88 |
| HLA-DRA | major histocompatibility complex, class II, DRα | 2.11 |
| HLA-DRB1 | major histocompatibility complex, class II, DRβ1 | 1.79 |
| HLA-E | major histocompatibility complex, class I, E | 1.96 |
| HLA-DPA1 | major histocompatibility complex, class II, DPα1 | 2.59 |
| HLA-DQB1 | major histocompatibility complex, class II, DQβ1 | 2.16 |
| HLA-DRB5 | major histocompatibility complex, class II, DRβ5 | 1.48 |
| HCP5 | HLA complex P5 | 1.36 |
| CIITA | class II, major histocompatibility complex, transactivator | 2.06 |
| CD74 | CD74 molecule | 1.78 |
| MICB | MHC class I polypeptide-related sequence B | 2.05 |
| **Free radical scavengers/DNA damage response** | | |
| DUOX1 | dual oxidase 1 | 2.02 |
| DUOX2 | dual oxidase 2 | 1.43 |
| GPX2 | glutathione peroxidase 2 (gastrointestinal) | 1.25 |
| GSTA1 | glutathione S-transferase α1 | -2.34 |
| GSTA2 | glutathione S-transferase α2 | -2.42 |
| MT2A | Metallothionein 2A | 2.56 |
| PARP14 | poly (ADP-ribose) polymerase family, member 14 | 1.48 |
| PRDX1 | peroxiredoxin 1 | 1.04 |
| SOD2 | superoxide dismutase 2, mitochondrial | 1.47 |
| XDH | xanthine dehydrogenase | 1.70 |
| **Apoptosis related** | | |
| BBC3 | BCL2 binding component, PUMA | 1.44 |
| BCL2A1 | BCL2-related protein A1 | 2.03 |
| BMF | Bcl-2 modifying factor | -0.43 |
| CFLAR | CASP8 and FADD-like apoptosis regulator, FLIP | 1.03 |
| FAIM3 | Fas apoptotic inhibitory molecule 3 | 2.38 |
| **Others** | | |
| BMP2 | bone morphogenic protein2 | 1.08 |
| CRABP2 | cellular retinoic acid binding protein 2 | 1.38 |
| FOLR1 | folate receptor 1 (adult) | -1.42 |
| GDF10 | growth differentiation factor 10 | -Inf |
| KCNK16 | potassium channel, subfamily K, member 16 | -1.39 |
| RASGRP1 | RAS guanyl releasing protein 1 (calcium and DAG-regulated) | 1.08 |
| REG1A | regenerating islet-derived 1α | -2.45 |
| REG1B | regenerating islet-derived 1β | -2.96 |
| REG3A | regenerating islet-derived 3α | -1.20 |
| REG4 | regenerating islet-derived family, member 4 | -2.12 |
| SKAP2 | src kinase associated phosphoprotein 2 | -0.72 |
| WNT5A | wingless-type MMTV integration site family, member 5A | 1.31 |
| WTAP | Wilms tumor 1 associated protein | 1.03 |

For a selected number of genes a comparison is shown between the 5 studied islet samples (see Table 1) cultured under control conditions and in the presence of cytokines (IL-1β + IFN-γ). The log_2_ of the proportion between the sum of the RPKM for all the transcripts from the same gene under cytokine treatment and the same sum obtained under control conditions was taken as measure of change in gene expression. A difference in gene expression was considered significant if the corrected p value <0.05. The table contains the median of the significant changes in gene expression. With a few exceptions genes were only taken up in the list when they were significantly changed in expression in one direction for at least 4 islet samples and changed in the other direction for none. JUNB and SH2B3 were significantly downregulated and BMF was significantly up-regulated in 1 islet preparation but they were added to this list for the sake of completeness.
